# Tuning the properties of peptide imprinted nanoparticles for protein immunoprecipitation using magnetic streptavidin beads

**DOI:** 10.1007/s00604-024-06782-7

**Published:** 2024-10-29

**Authors:** Ainhoa Elejaga-Jimeno, Alberto Gómez-Caballero, Gontzal García del Caño, Nora Unceta, Miquel Saumell-Esnaola, Joan Sallés, M. Aránzazu Goicolea, Ramón J. Barrio

**Affiliations:** 1 Department of Analytical Chemistry, University of the Basque Country UPV/EHU, 01006 Vitoria-Gasteiz, Spain; 2 Department of Neurosciences, Faculty of Pharmacy, University of the Basque Country UPV/EHU, 01006 Vitoria-Gasteiz, Spain; 3 Department of Pharmacology, Faculty of Pharmacy, University of the Basque Country UPV/EHU, 01006 Vitoria-Gasteiz, Spain; 4 Bioaraba, MetaboloMIPs, 01008 Vitoria‑Gasteiz, Spain; 5 Bioaraba, Neurofarmacología Celular y Molecular, 01008 Vitoria‑Gasteiz, Spain; 6 Centro de Investigación Biomédica en Red de Salud Mental (CIBERSAM), 28029 Madrid, Spain

**Keywords:** Molecularly imprinted nanoparticles, Epitope imprinting, Thermoresponsive polymers, Artificial antibody, Immunoprecipitation, CB_1_ receptor

## Abstract

Maximizing the binding properties of thermoresponsive molecularly imprinted nanoparticles (MIN) was aimed to explore their feasibility as antibody substitutes in protein immunoprecipitation (IPP) with magnetic streptavidin beads (MSB). Thermoresponsive MIN targeting the cannabinoid CB_1_ receptor were produced by epitope imprinting through solid-phase synthesis. It was intended to determine how different variables influenced physicochemical features, binding behaviour and immunoprecipitation of the target recombinant glutathione S-transferase tagged fusion protein (GST-CTer). Such variables included the cross-linking degree of MIN, and variables like pH, temperature or the use of Tween-20 for binding and IPP experiments. The cross-linker (CL) amount influenced the coil-to-globule transition of thermoresponsive MIN, making the lower critical solution temperature (LCST) decrease from 37.2 °C using 5% of CL, to 29.0 °C using 25%, also suggesting higher plasticity on the former. Temperature influence on size was corroborated by dynamic light scattering, observing size reductions from 250–450 nm (RT) to 70–100 nm (> LCST) for MIN produced with 5–15% of CL. However, binding behaviour did not clearly  improve for more than 10% CL. Further experiments revealed that temperature and pH control were critical for efficient binding and release, selecting 40 °C and pH 5 as appropriate. Following binding experiments, the GST-CTer-MIN complex was successfully immunoprecipitated using MSB, achieving an IPP efficiency of 11.48% over the initial input protein concentration, which was calculated after SDS-PAGE separation and Western blot analysis. The methodology may be exploited for selective protein extraction and quantification from complex tissue homogenates.

**Graphical Abstract:**

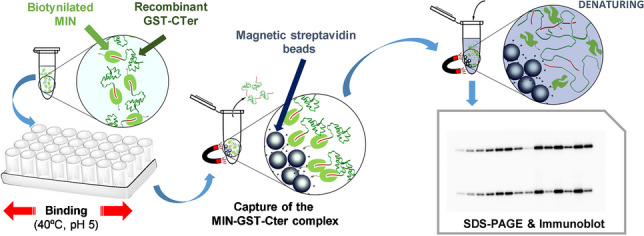

**Supplementary Information:**

The online version contains supplementary material available at 10.1007/s00604-024-06782-7.

## Introduction

Molecularly imprinted nanoparticles (MIN) are synthetic smart nanomaterials which pretend to imitate the binding behaviour of their natural counterparts, like antibodies or enzymes [1]. Even if natural receptors have excellent recognition capabilities, they present inherent limitations due to their biologic nature, showing poor stability under non-physiological conditions. In fact, pH and temperature variations may induce conformational changes on them, thus losing their functionality. MIN, as artificial antibodies, are intended to overcome common drawbacks associated with natural ones, showing improved stability to high temperatures, extreme pH and pressure, which allows them for being stored for long time periods even at room temperature [2]. Furthermore, MIN present higher batch-to-batch reproducibility, and relatively faster and more cost-effective production [3]. In addition, production of imprinted materials does not involve experiments with animals, which may contribute to a significant reduction of their use. Given their benefits and potential applicability, it is expected that, in the future, molecularly imprinted polymers (MIP) may replace many biomolecules, not only antibodies, but also enzymes or DNA [4].

Over the last decades, the fabrication of nano-sized molecularly imprinted polymers (MIP) has experienced swift growth due to their benefits over larger sized imprinted polymers, including a higher surface-to-volume ratio, which, in turn, contributes to an increase of the number of binding sites with better accessibility for the target ligand, thereby reducing mass transfer kinetics. All these attributes make MIN especially interesting for binding macromolecules such as proteins, which suffer from slow binding kinetics upon high cross-link densities [5], being a promising tool for in vivo applications including virus counteracting [3], toxin scavenging [6] and drug delivery [7]. The remarkable binding performance shown by MIN towards proteins has definitely widened MIN applications in biomedicine, being recently focused on targeting surface receptor proteins expressed on cancer cells [8], such as the epidermal growth factor receptors (EGFR) [9] or the vascular endothelial growth factor (VEGF) [10].

Maximising binding affinity and reducing cross reactivity of imprinted nanoparticles plays a key role if real antibody substitutes are to be produced for the application in the biomedical field. In this regard, fundamental research on imprinting technology is crucial to generate the basic knowledge required to achieve those ideally perfect plastic receptors. Thus, the influence of the polymerisation conditions, such as monomer composition [11], cross-linker density [12, 13] or polymerisation time [14], on binding parameters must be determined when MIN with highest binding performance are desired. Within the polymerisation mixture, functional monomers are responsible for establishing non-covalent interactions with the template, which could be a linear peptide [15], a motif [3] or a whole globular protein [16, 17] when MIN targeting proteins are produced [5]. By contrast, cross-linker monomers are the ones providing physical stability to the formed non-covalent complex, having a significant effect on the physical properties of the resulting polymers and little contribution to specific interactions that may occur within the binding site [5]. In addition, the amount of cross-linker directly determines the rigidity of produced nanoparticles, and it is undoubtedly recommendable to determine the right balance of flexibility/rigidity to produce nanoparticles with improved binding performance [13]. High percentages of this constituent give rise to too rigid materials, hindering accessibility of target ligands to imprinted sites and decreasing binding affinity [12, 13]. However, high cross-linker densities contribute to selectivity improvement, as the spatial stability of binding sites increases, which reduces cross-reactivity. In any case, based on the above, more research is still needed in order to determine the optimal cross-linking density for the production of MIN that resemble more to real antibodies [12].

Over the last decade, the imprinting of polymers that undergo conformational and structural changes upon external stimuli (such as temperature, pH or light) has grown considerably [18, 19]. Particularly, polymers made of monomers such as N-isopropyl acrylamide (NIPAm) and/or N-tert-butylacrylamide (TBAm) are known for being thermoresponsive [15, 20], being capable to swell or shrink upon temperature change. Poly(NIPAm) presents a lower critical solution temperature (LCST) of about 32 °C, at which the degree of swelling of the polymer particles changes undergoing a volume phase transition from a random-coil hydrophilic structure to a more hydrophobic globular structure, which repels water and, thus, shrinks [21, 22]. Liu et al. reported that the coil-globule transition process of single chain poly(NIPAm) was divided into three steps named nucleation, adjacent mergence and bead stacking (NAS transition), finding that the final globule was composed of stacked beads rather than being a uniform compact globule [21]. This unique feature makes these materials especially interesting for protein imprinting, as temperature-controlled ligand binding-release may be done, favouring ligand access to or from imprinted sites. In this regard, thermoresponsive MIN have been reported as artificial antibodies for proteins such as lysozyme [23], thyroid peroxidase [24], bovine haemoglobin [25], trypsin [26, 27], human serum albumin [28] or the cannabinoid receptor type 1 (CB_1_ receptor) [15]. On the other hand, MIN ability to respond to thermal stimuli also helps in conducting temperature-based nanoparticle purification by affinity chromatography after MIN production, which allows for discarding low affinity nanoparticles and collecting the ones with highest affinity [29]. This purification has been used after solid-phase synthesis of imprinted nanoparticles targeting proteins such as trypsin and/or kallikrein [27, 30, 31], or the CB_1_ receptor [15]. Here, MIN are produced by solid-phase synthesis using the epitope imprinting approach. Thereafter, an affinity-based washing is conducted at temperatures above the LCST first, to remove low affinity nanoparticles, and later, below LCST, to dissociate the MIN-peptide complex obtaining a suspension of high affinity nanoparticles.

Thermoresponsiveness of MIN depends not only on the type of monomers used for polymerisation, but also on the cross-linker amount over functional monomers. Ambrosini et al. produced thermoresponsive MIN using a 95/5 molar ratio of NIPAm and N,N-ethylenebis(acrylamide) (BisAm) [27]. Higher percentages of BisAm (10–20%) produced nanoparticles which did not change size upon temperature change, thereby not showing thermoresponsivity [27, 30]. Navarro et al. reported the influence of the cross-linking density on thermoresponsive nanogels, and they did not observe any clear change on cloud point temperature (i.e. the temperature of the inflection point of the normalised transmittance at 500 nm vs temperature curve) of nanogels prepared with different cross-linker densities, which were synthesised using cross-linker:NIPAm ratios that varied between 25/75 and 50/50. However, the nanogels with the highest cross-linking degree suffered much less size change upon heating than nanogels prepared with less cross-linker*.* This may happen due to an increase of the rigidity of the system on more cross-linked materials, which also provide a lower swelling ratio [32]. Based on all the above, it is undoubtedly crucial to determine how the cross-linking amount in a particular monomer mixture influences thermoresponsive character on the resulting polymer nanogel. Besides, research is still needed to determine how all this temperature-based rearrangement and coil-to-globule transition phenomena contribute to binding behaviour of imprinted nanomaterials. Going deeper into this knowledge may pave the way for the creation of MIN with faster kinetics and better binding affinities, approaching even more to the behaviour of natural receptors.

This work aims to explore how the cross-linker density influences physicochemical characteristics and binding behaviour of peptide imprinted nanogels. Based on previous experience of our research group on solid-phase synthesis of thermoresponsive anti-CB_1_ artificial antibodies [15], we wanted to go further on basic knowledge concerning this methodology to determine how temperature and cross-linking densities of produced MIN may compromise specific binding of the recombinant fusion GST-CB1 protein. Increasing binding affinity and selectivity may widen the applicability of these anti-CB_1_ antibodies to other (bio)analytical assays where complex biological matrices are targeted. To this end, a series of nanoparticles have been produced here using variable cross-linker ratios versus the total monomer moles added to the polymerisation mixture. MIN have been fabricated by solid-phase synthesis using a 15 amino acid sequence (458-KVTMSVSTDTSAEAL-472) as template. This sequence matches with the extreme end of the cytosolic carboxy-terminal (C-terminal) tail of the CB_1_ receptor, which is the mostly expressed G-protein coupled receptor (GPCR) in the mammalian central nervous system, being especially abundant in the human brain [33]. Dysregulation of CB_1_ receptor-mediated signalling may contribute to several pathological conditions, including depression, anxiety, epilepsy, schizophrenia or neuropathic pain [34, 35]. Given the physiopathological significance of the neuronal CB_1_ receptor signature and the low availability of effective and high affinity natural antibodies for this purpose [36], the production of artificial antibodies is presented here as a tool to broaden the knowledge regarding this receptor. All the findings of this research have been exploited to widen the applicability of artificial MIP-based antibodies, tested here as natural antibody substitutes for immunoprecipitation assays using streptavidin-magnetic beads, which are a versatile tool that enables the pulling down compounds labelled with biotin. Produced fit-for-purpose biotin-labelled MIN have proven effective for immunoprecipitating a recombinant GST-CB_1_ fusion protein, which may be exploited in the future for the purification, enrichment and determination of the concentration of this target receptor on heterologous cell expression systems and brain tissue homogenates.

## Experimental

### Materials and reagents

Glass beads of 150–212 μm in diameter were purchased from Merck (Spain). The silanes (3-aminopropyl)triethoxysilane (APTES) (99%) and 1,2-bis(triethoxysilyl)ethane (BTESE) (95%), and the monomers N-isopropylacrylamide (NIPAm) (97%), N-tert-butylacrylamide (TBAm) (97%), acrylic acid (AA) (99%), N-(3-aminopropyl) methacrylamide hydrochloride (APMA) (98%) and N,N′-methylenebis(acrylamide) (BisAm) (99%) were also obtained from the same company. Succinimidyl iodoacetate (SIA) (> 97%) was acquired from Thermo Fisher Scientific (Spain).

The water-soluble photoiniferter diethylthiocarbamoylsulfanyl acetic acid (DCAA) was synthesised using sodium diethyldithiocarbamate (98%) and sodium chloroacetate (98%) acquired from Alfa-Aesar (Spain) (yield 67.92%), as described in [15]. Synthesised polymer nanoparticles were biotinylated using ( +)-biotin N-hydroxysuccinimide ester (biotin-NHS) (98%) purchased from Fisher Scientific (Spain). The fluorophore Alexa Fluor 488 C5-maleimide used for peptide labelling was also purchased from this company.

The target peptide (purity 96.85%, MALDI-TOF (m/z): (M + H^+^) 1643.24) containing an additional cysteine residue (C-KVTMSVSTDTSAEAL) was custom-synthesised by Caslo ApS (Denmark). The specified sequence matches with the carboxy-terminal sequence of the CB_1_ endocannabinoid receptor.

For immunoprecipitation experiments, Eppendorf Protein LoBind tubes were used, and Tween-20 was purchased from Merck (Spain). Streptavidin magnetic beads were acquired from Thermo Fisher Scientific (Spain). Urea-denaturing buffer comprised 20 mM Tris–HCl, pH 8.0, 12% glycerol, 12% urea, 5% DTT, 2% SDS and 0.01% bromophenol blue. Acetic acid used for this assay was obtained from Panreac (Spain). Immun-Blot PVDF membranes and Clarity Western ECL Substrate were acquired from Bio-Rad (Spain), while Albumin Bovine Fraction V (BSA) MB Grade and dry milk powder used for the blocking buffer were purchased from Nzytech (Portugal). As anti-CB_1_ antibody, an immunoaffinity-purified rabbit polyclonal antibody (CB1-ImmGs; ImmunoGenes Kft.) was used, and as secondary antibody a horseradish peroxidase conjugated donkey anti-rabbit IgG was employed (NA934; Amersham Biosciences).

HPLC-grade solvents such as methanol (MeOH), acetonitrile (ACN), acetone and ethanol (EtOH) were acquired from Scharlab (Spain), and dimethyl sulfoxide (DMSO) was acquired from Panreac (Spain). All buffer solutions were formulated with ultrapure water (resistivity of 18.2 MΩ cm), which was achieved with Elix20 reverse osmosis and Milli-Q water purification systems from Merck (Spain). All other reagents were of analytical grade and were used without further modification.

### Apparatus

Molecularly imprinted nanoparticles were synthesised by UV initiated photopolymerisation using two Summer Glow HB175 (75 W) UV lamps from Hapro (The Netherlands). Turbidity assays were conducted on a Cary 60 UV–Vis spectrophotometer provided with a Peltier thermostatted single cell holder connected to a water circulation bath. For these experiments, the cell holder was set to the desired temperature, and absorbance was monitored until signal stabilisation.

Size analysis and zeta potential measurements of produced nanoparticles were performed using a Zetasizer Ultra instrument from Malvern Panalytical (Spain). SEM images were acquired using a Schottky-type field emission scanning electron microscope model JSM-7000F (JEOL, Tokyo, Japan). Samples were dried on a metallised cover glass, and, then, they were sputter coated with gold. X-ray photoelectron spectroscopy (XPS) measurements were conducted using a Versaprobe III Physical Electronics (ULVAC) system with an Al Kα (1486.7 eV) monochromatic radiation source. The XPS spectrometer energy scale was calibrated using Ag 3d5/2 photoelectron line located at 368.26 eV. Acquired spectra were processed using the software CasaXPS 2.3.26.

Gel filtration chromatography (GFC) experiments were conducted using an Agilent Technologies 1260 series liquid chromatograph consisting of a binary pump, a vacuum degasser and a Rheodyne manual injector with a 20 μL loop. The system was coupled to a fluorescence detector of the 1200 series. For system condition control and data analysis, the Agilent LC ChemStation software (Agilent Technologies, USA) was employed. A PolySep-GFC-P 2000 column from Phenomenex (Spain) with an internal diameter of 7.8 mm and a length of 300 mm served as the stationary phase, which was installed together with a PolySep-GFC-P guard column. This column is recommended for the separation of water-soluble macromolecules in the range of 100 Da–10 kDa. A 50:50 mixture of 0.1 M ammonium formate and methanol was used as mobile phase (flow rate: 0.5 mL min^−1^). Fluorescence signals were recorded using excitation and emission wavelengths of 495 nm and 528 nm.

Apparatus used for the electrophoresis and transference of the samples to PVDF membranes were Protean II xi cell and Trans-Blot transfer system, respectively, both from Bio-Rad (USA). For the acquisition of the images, ChemiDoc MP Imaging System, also from Bio-Rad, was used.

### Production of imprinted and control nanoparticles

Production of imprinted and non-imprinted nanoparticles, used as control, has been included in SI.

### Nanoparticle characterisation

Particle size and zeta-potential were determined by dynamic light scattering (DLS) (*n* = 3). For size analysis, nanoparticle suspensions having a concentration of 100 mg L^−1^ were prepared in 25 mM formate buffer (pH 3) to avoid particle aggregation. DLS measurements were done at three different temperatures, below LCST, at LCST and above the LCST. These experiments allowed for comparing sizes of the different MIN at different conditions, to better understand the influence of the cross-linker percentage on each produced nanoparticle batch. Zeta-potential measurements were performed at LCST for 100 mg L^−1^ nanoparticle suspensions prepared in 25 mM formate (pH 3 and pH 5), phosphate (pH 7) or carbonate buffers (pH 9).

Biotinylated and non-biotinylated imprinted (MIN) and non-imprinted nanoparticles (NIN) were also analysed by XPS to get more information concerning their functionalities. To this end, *n* = 3 synthesis batches of MIN or NIN suspensions were mixed to get enough solid material for XPS analysis. Then, they were desalted, washed with ultrapure water and concentrated down to ≈ 1.5 mL using Amicon Ultra centrifugal filters. Finally, water was evaporated to dryness under a N_2_ stream, using a sample concentrator apparatus working at 45 °C. Resulting solid MIN or NIN samples were subjected first to wide scan analysis (step energy 0.2 eV, pass energy 224 eV) to determine the elements present on each sample. Then, a high-resolution analysis of detected elements was performed (detail scan: step energy 0.05 eV, pass energy 27 eV, time per step 20 ms) with an electron emission angle of 45°.

### Binding experiments

Binding affinity of produced nanoparticles was determined by batch rebinding experiments. To this end, the target peptide (C-KVTMSVSTDTSAEAL) was labelled with the fluorophore Alexa Fluor 488 C5-maleimide as detailed in SI. Peptide labelling allowed for the sensitive determination of the free (unbound) peptide concentration by gel filtration chromatography coupled to a fluorescence detector (GFC-FD) after binding experiments.

Preliminarily, we wanted to know the conditions at which MIN provided the highest binding over NIN used as control. For this, a series of 10^−8^ M solutions of the labelled peptide were incubated with 0.5, 1, 2.5, 5, 10, 50, 100, 150, 175 and 200 µg mL^−1^ of MIN for 24 h in order to assure MIN-peptide binding equilibrium. These experiments were conducted in triplicate (*n* = 3) using 25 mM formate (pH 5) or 25 mM phosphate buffer (pH 7.4) as binding media, both at RT and 40 °C. After incubation, all solutions were centrifuged at 10,000 rpm for 30 min, and the supernatant was collected for further analysis via GFC-FD. Chromatographic separation of the labelled peptide was required to properly discriminate the fluorescence signal coming from the labelled peptide and the signal coming from the remaining excess of the labelling reagent that always exists to a greater or lesser extent in these solutions.

Once having optimised the binding protocol, batch rebinding experiments were conducted also in triplicate (*n* = 3) at selected conditions. For this, a fixed concentration (150 mg L^−1^) of either MIN or NIN particles was incubated in the presence of increasing concentrations of the peptide, that is, 10^−9^, 2.10^−9^, 4.10^−9^, 6.10^−9^, 8.10^−9^, 10^−8^, 2.10^−8^, 4.10^−8^, 6.10^−8^, 8.10^−8^ and 10^−7^ M prepared in 25 mM formate buffer (pH 5). After 24 h, free (unbound) peptide was quantified in the supernatant by GFC-FD. From the experimental binding isotherm, the equilibrium dissociation constant (*K*_*D*_) was determined after fitting the curve to a one-to-one Langmuir binding model using the software GraphPad Prism. Kinetic assays were also performed for produced nanomaterials following the same procedure as detailed above (*n* = 3). Here, four different peptide solutions at concentrations of 10^−9^, 10^−8^, 5.10^−8^ and 10^−7^ M were incubated with 150 mg L^−1^ of MIN for 30 min, 1 h, 2 h, 4 h, 6 h, 8 h, 12 h, 16 h, 20 h and 24 h.

### Anti-CB_1_ nanoparticles as antibody substitutes for immunoprecipitation experiments

**Fig. 1:**
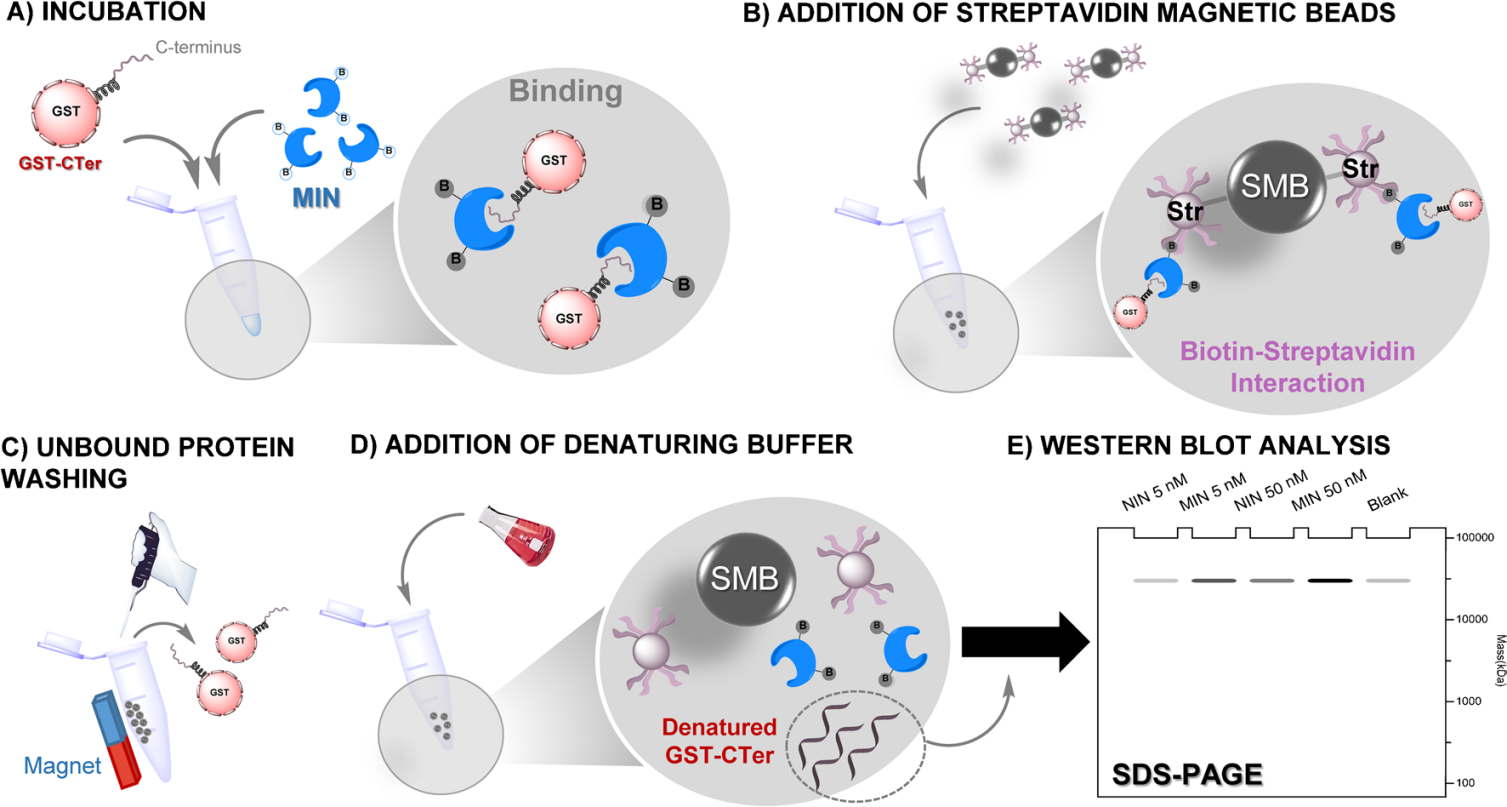
Schematic representation of the immunoprecipitation procedure using anti-CB_1_ MIN

All these experiments were carried out using the recombinant fusion protein GST-CB1_414-472_ (GST-CTer) produced as described in [15]. Initially, prior to immunoprecipitation (IPP) assays, *n* = 3 replicates of a series of blank experiments were carried out using 500 µL of 100 nM solutions of the target protein prepared in different buffers. As buffers, 25 mM phosphate buffer (PB) and 25 mM phosphate buffered saline (PBS) (0.15 M NaCl) were used to work at pH 6 or 7, whereas 25 mM formate (FB) and 25 mM formate buffered saline (FBS) were selected for pH 5. The protein solutions were added to 1.5 mL Eppendorf tubes and incubated in a thermomixer R apparatus (Eppendorf) at 40 °C for 24 h under continuous orbital shaking (950 rpm). Thereafter, the pH of all solutions was adjusted to 7, and 5 µg of streptavidin magnetic beads (SMB) was added, incubating the mixture for 1 h at 40 °C. After binding, the beads were collected with a magnet and washed three times using either 500 µL of 25 mM PB (pH 7) or PB-T (0.05% Tween-20) at 40 °C, discarding the supernatant. The beads were finally resuspended in 50 µL of a urea-denaturing buffer to release and denature bound GST-fusion proteins, and then tenfold diluted. Finally, 15 µL of the prepared dilutions was resolved by SDS–polyacrylamide gel (SDS-PAGE) electrophoresis (Fig. 1).

For IPP assays (*n* = 3), 500 µL suspensions of MIN or NIN were prepared in 25 mM FBS (pH 5), including increasing concentrations of the GST-CTer target (5, 10, 25 and 75 nM) and a fixed amount of biotinylated MIN or NIN to reach a nanoparticle concentration of 0.015 mg mL^−1^. These mixtures were incubated at 40 °C for 24 h under orbital shaking, then the pH was adjusted and SMB were added, as detailed for the blank experiments above. After that, the beads were washed three times using 500 µL of 25 mM PB (pH 7) at 40 °C, discarding the supernatant. Remaining MIN-SMB were resuspended in 50 µL of urea-denaturing buffer, and tenfold diluted prior to SDS-PAGE analysis. These mixtures are referred to as immunoprecipitation (IPP) samples.

In the first lanes of the SDS-PAGE gels, 15 µL of different standard solutions of the target GST-CTer protein was loaded to reach 25, 50, 100 and 200 fmol of GST-CTer in each lane. The remaining lanes were loaded with diluted IPP samples of MIN and NIN after immunoprecipitation experiments with increasing concentrations of GST-CTer. Protein migration was conducted at 100 V using a Protean II xi cell. After electrophoresis, proteins were transferred to PVDF membranes at 4 °C and 30 V overnight. Subsequently, they were gently washed with ultrapure water, and proteins were fixed by incubation in a 7% acetic acid and 10% methanol aqueous solution for 15 min. PVDF membranes were washed with water for 10 min and then with 0.2 M PBS containing 0.1% Tween-20 (PBS-T). Thereafter, the membranes were incubated for 1 h in blocking buffer (0.1 M PBS, pH 7.4, 0.5% BSA, 5% of dry milk powder and 0.2% Tween-20) followed by overnight incubation at 4 °C in a 0.2 µg mL^−1^ solution of the anti-CB_1_ antibody prepared in blocking buffer. After three 10-min washings with PBS-T, the membranes were incubated for 1 h at RT with a HRP-conjugated donkey anti-rabbit antibody 1:10,000 diluted in blocking buffer. Finally, PVDF membranes were washed three times in PBS-T to remove unbound secondary antibodies, and protein bands were developed by chemiluminescence with the Clarity Western ECL Substrate from Bio-Rad (Spain), visualised and acquired with a ChemiDoc MP Imaging System (Bio-Rad).

## Results and discussion

### Characterisation of nanoparticles

**Fig. 2:**
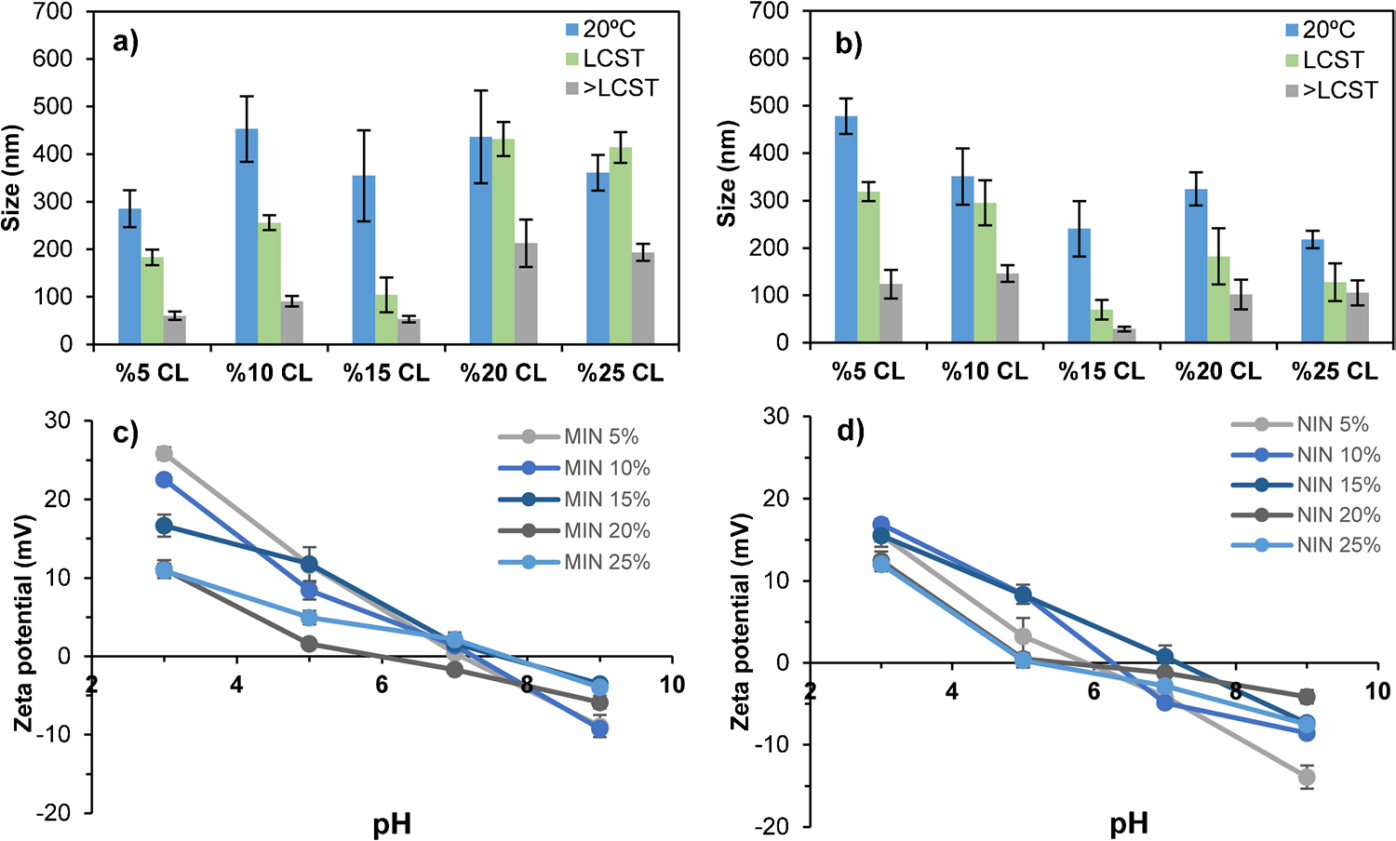
Particle sizes determined by DLS at 20 °C, at the LCST and over LCST for **a** MIN and **b** NIN produced using different percentages of cross-linker, and zeta potential values registered for the same **c** MIN and **d** NIN nanoparticles. Error bars describe ± SD of *n* = 3 measurements of each MIN or NIN sample

Considering the thermoresponsive character of produced nanomaterials discussed in SI, size analysis was conducted at three different temperatures, below LCST (20 °C), at estimated LCST (Table S1) and above LCST (50 °C), as described in Sect. 2. We wanted to know if the cross-linker percentage used for polymer synthesis contributed in any extent on the final particle size of the produced nanomaterial. We hypothesised that larger amounts of cross-linker may give rise to bigger sizes, and less surface charge, due to a lower proportion of ionisable functional groups coming from functional monomers such as acrylic acid or APMA. In order to corroborate all this, DLS analyses were carried out for MIN and NIN nanoparticles produced with 5%, 10%, 15%, 20% and 25% of BisAm as cross-linker. Obtained results are depicted in Fig. 2a, b. It can be deduced from the charts that both MIN and NIN particles presented biggest sizes when working at 20 °C, having sizes ranging from about 250 to 450 nm. This higher size may be explained as they are in their expanded coil state. When setting higher temperatures than 20 °C, the size decreased as a result of the coil to globule transition. As a rule, particles were smaller at LCST and even smaller at temperatures above LCST. Focusing on MIN (Fig. 2a), sizes were quite similar working at temperatures > LCST and using 5, 10 or 15% of CL, being around 80–100 nm. However, using higher percentages, that is, 20 or 25%, the size increased considerably to around 200 nm. This size increase was observed when working at LCST (green bar) or > LCST (grey bar). This phenomenon may be happening for two reasons; as a result of more cross-linker in the polymerisation mixture, or due to the formation of particle aggregates in the MIN suspension. Higher cross-linker percentages may presumably had reduced surface charge of produced nanoparticles, as less ionisable groups may be present per particle surface unit. Therefore, interparticle charge repulsion forces would be minimised, which may influence negatively colloidal stability of the MIN suspension. Aggregation at 20 °C was not so evident, as it can be deduced from the blue bar of Fig. 2a. This could arise because MIN are in coil state, which is known to be more hydrophilic, thereby showing less tendency to aggregate. Unlike for MIN, the aggregation tendency for NIN at high cross-linker percentages was not so clear. One possible explanation for this may come from the fact that ionisable groups coming from functional monomers will likely be randomly distributed throughout the entire NIN particle providing higher colloidal stability, whereas for MIN, ionisable groups will predominantly be present in binding sites inside the particle, which may have negative influence on particle repulsion giving rise to lower stability. These assumptions were further corroborated through zeta potential analyses (Fig. 2c, d). In all cases, MIN nanoparticles presented a slightly higher zeta potential values than NIN nanoparticles at pH 3, which may be indicative of more positively charged particles due to the presence of a larger number of amino groups coming from the APMA monomer. The peptide used as template has three amino acids (C-KVTMSVST**D**TSA**E**A**L**) with carboxylic groups, which are expected to induce the incorporation of functional monomers with amino groups to the particles being synthesised. This would allow for obtaining particles with higher positive charge, and, therefore, higher zeta potential at acidic pH. Zeta potential also decreases as the CL% is higher. This may be attributable to a lower percentage of APMA monomers within each particle as the CL amount is higher. On the other hand, NIN particles present a slightly lower zeta potential at high pH, which may be related with a higher presence of acrylic acid, especially for NIN produced with 5% CL. Acrylic acid is the only monomer bearing –COOH groups; thus, it is expectable that negative charge at basic pH may come from it.

**Fig. 3:**
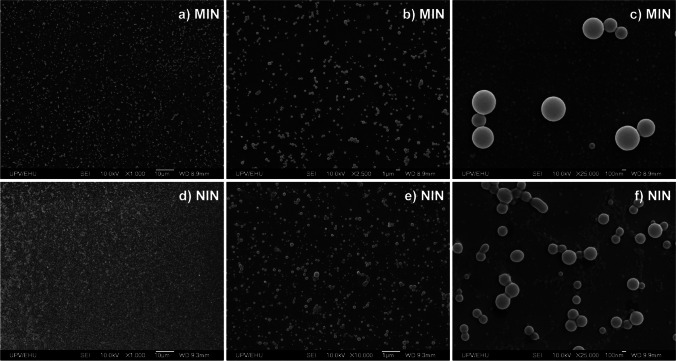
SEM micrographs obtained at different magnifications for **a** MIN and **b** NIN

Morphological characterisation of produced nanoparticles was also carried out by SEM analysis. Figure 3 depicts SEM micrographs obtained at different magnifications for MIN and NIN nanoparticles synthesised using 10% of CL. Spherical and regular nanoparticles were observed in all cases, being NIN nanoparticles slightly smaller, which may be in accordance with results obtained from DLS analysis.

**Fig. 4:**
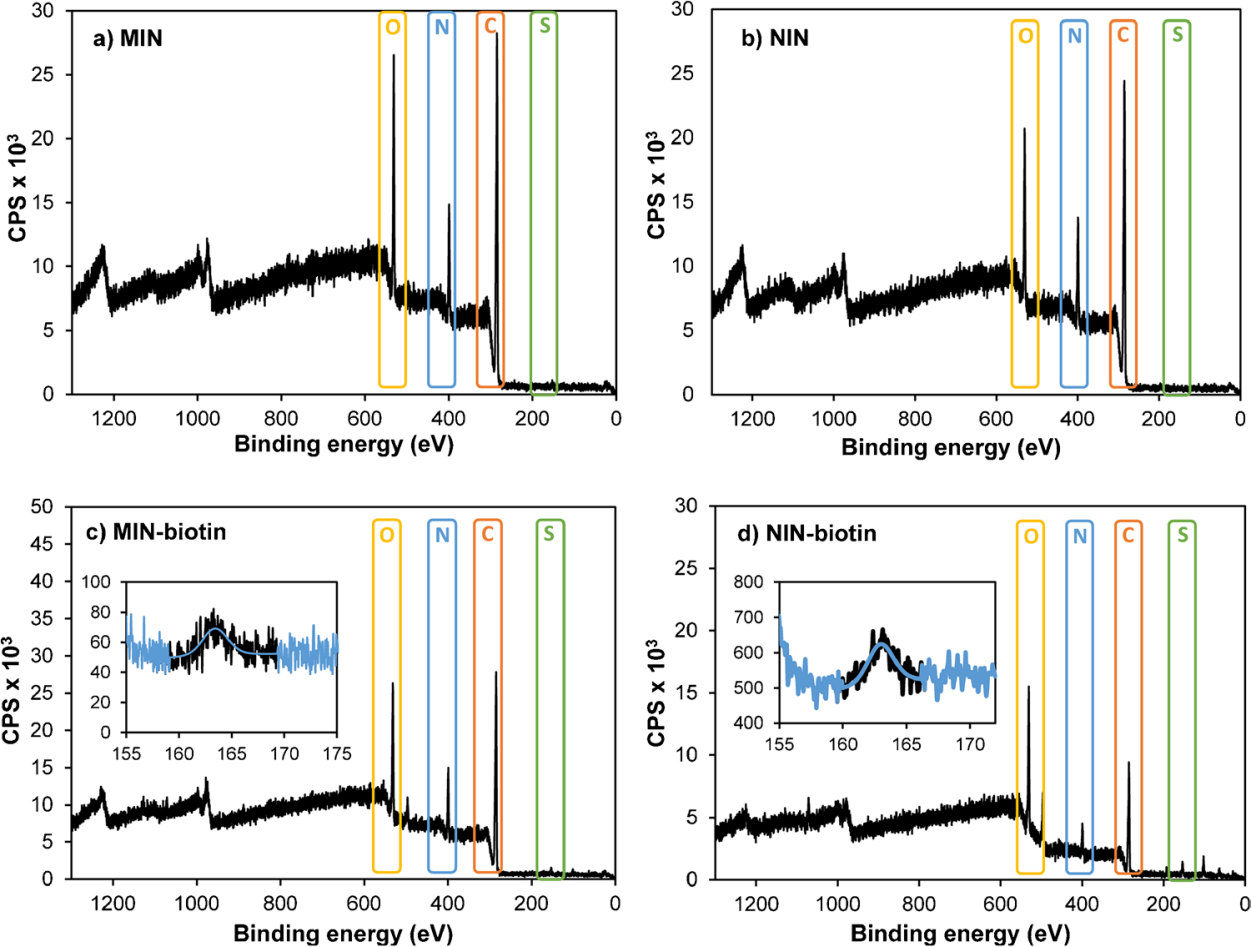
Survey XPS spectra for **a** MIN, **b** NIN, **c** biotinylated-MIN and **d** biotinylated-NIN

Chemical functionalities of MIN and NIN materials produced with 10% of CL were examined by XPS, to determine whether the presence of the template during polymerisation favoured any change in monomer composition on MIN nanoparticles. Survey spectra of MIN and NIN spectra were very similar (Fig. 4a, b). However, in spectra corresponding to biotinylated nanoparticles, an additional small peak at 163.4 eV (S 2p) can be distinguished (Fig. 4c, d). Upon closer examination of the detailed spectra (Fig. 4c, d insets), the peaks, although in close proximity to the background noise signal, were more clearly visible, confirming the presence of sulphur coming from biotin and suggesting successful nanoparticle biotinylation. Proper biotinylation of produced nanoparticles was further corroborated with the Pierce Biotin Quantitation Kit (Thermo Scientific, USA), as detailed in SI (Table S2). Wide scan spectra of tested nanoparticles show intense C1s peaks for all samples (Fig. S3). The detailed spectra of C1s showed a consistent pattern in all measurements, with three peaks attributed to distinct carbon atoms within the polymer structure. Particularly, the peaks at 284.6 eV, 285.8 eV, and 287.5 eV corresponded to carbon atoms in C–C/C–H, C–N/C–O, and N–C = O/O–C = O groups, respectively. Additionally, oxygen (520–540.7 eV area) and nitrogen (389.7–409.8 eV area) peaks were consistently present in all samples, which did not clearly reveal any difference in monomer proportions between MIN and NIN. Obtained results are summarised in Table S3.

### Binding experiments

In order to study the binding event between imprinted nanoparticles and their ligand, the target peptide was labelled with a fluorescent dye (Alexa Fluor 488) so that a fluorescence detector could quantify its free concentration in different solutions using a chromatographic system. In the beginning, a series of experiments at different temperatures and pH were conducted in order to determine the conditions at which MIN-peptide interaction is maximised. In this regard, and considering nanoparticles’ ability to respond to temperature changes, two different temperatures (RT and 40 °C) were tested using formate (pH 5) or phosphate buffers (pH 7.4) as media; pH values at which both the peptide (isoelectric point: 3.9) and MIN are expected to be ionised, thereby favouring ionic interactions. All these experiments were carried out as described in Sect. 2, using solutions of 10^−8^ M of the labelled peptide containing increasing concentrations of MIN from 0.5 to 200 µg mL^−1^. The mixtures were incubated for 24 to ensure MIN-peptide binding equilibrium was reached, as it was later demonstrated with kinetic experiments depicted in Fig. S4. Thereafter, unbound (free) peptide concentration present in these solutions was quantified by GFC-FD. Bound percentage was calculated using the difference between measured free concentration and total concentration (10^−8^ M). Fig. S4a depicts bound percentages of the ligand as a function of MIN concentration. As it can be observed, little binding was observed at RT, even for highest MIN concentrations, which may be attributed to the expanded state that MIN may experience at RT. Additionally, pH 7 was not found appropriate for binding, even at 40 °C, which may be due to a lower stability of the suspension, contributing to particle aggregation and reduced binding. Thus, only 40 °C and pH 5 provided proper binding, regardless this binding occurred specifically at binding sites or not. To clarify this, binding results of the MIN were compared with non-imprinted ones, used as control, to determine how NIN nanoparticles may bind the target ligand through non-specific binding (Fig. S4b). Bound percentages of the ligand were much lower for NIN nanoparticles, and binding was only appreciated at a concentration level of 200 µg mL^−1^, being negligible for lower levels. These results were surprisingly positive, as they suggested that binding at MIN happened preferably through created binding sites rather than with the polymer matrix. Next, binding behaviour of the different MIN formulations was studied at pH 5 and 40 °C. No significant difference was observed between tested samples (Fig. S5), being bound percentage slightly higher for MIN synthesised using 20% of CL. Higher percentages than this were very close to solution gelation and did not provide a remarkable improvement over MIN synthesised with a 10% percentage. Moreover, as described before, particle aggregation is more likely to occur for MIN synthesised with a higher CL percentage. Therefore, 10% was selected for further experimental.

**Fig. 5:**
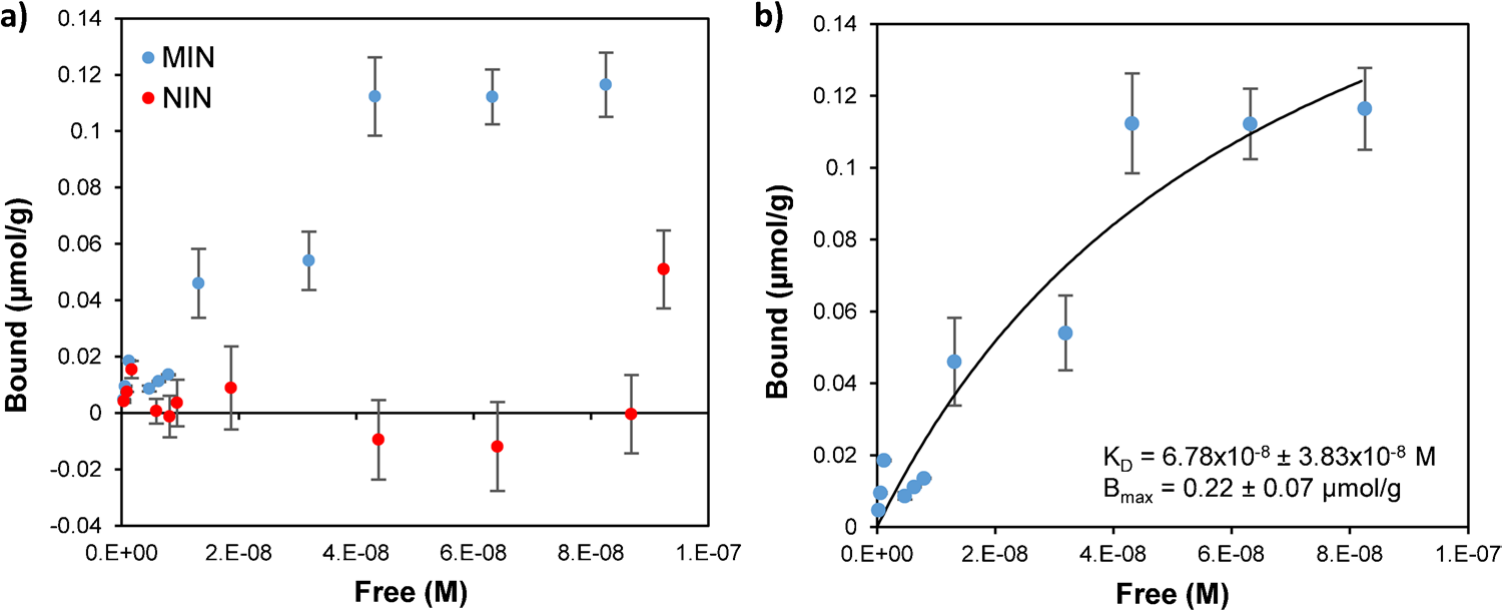
**a** Experimental binding isotherms showing dependence between bound and free peptide concentrations for MIN and NIN nanoparticles. **b** Experimental binding isotherm of the MIN fitted to a Langmuir model. Error bars describe ± SD of *n* = 3 independent rebinding experiments in both figures

Experimental isotherms obtained with MIN and NIN nanoparticles prepared with 10% of CL were constructed to determine binding parameter for this material (Fig. 5). The curves were then fitted to a Langmuir model, which assumes a single homogeneous binding site for each particle. The *K*_*D*_ (equilibrium dissociation constant) and *B*_*max*_ (maximum binding capacity) values obtained from the fitting were found to be (6.78 ± 3.83) × 10^−8^ M and 0.22 ± 0.07 µmol g^−1^, respectively (Fig. 5b). It is worth noting that affinity is in the nM level, close to that reported for natural antibodies.

### Application of anti-CB_1_ nanoparticles as antibody substitutes for protein immunoprecipitation

MIN synthesised using 10% of CL were tested in immunoprecipitation (IPP) experiments against the GST-CTer fusion protein to check the utility of these nanoparticles as potential substitutes of natural anti-CB_1_ antibodies for protein extraction, thereby expanding the applicability of imprinted nanomaterials in the bioanalytical field. These experiments are based on the use of magnetic beads with streptavidin attached to their surface (SMB). First, biotinylated MIN are exposed to the target GST-CTer for binding (step a) (Fig. 1), and, then, they are incubated with SMB (step b). Finally, the GST-CTer-MIN-SMB complex is pulled down with a magnet via the biotin-SMB interaction. After washing away unbound protein, remaining bound GST-CTer is denatured for Western blot analysis.

**Fig. 6:**
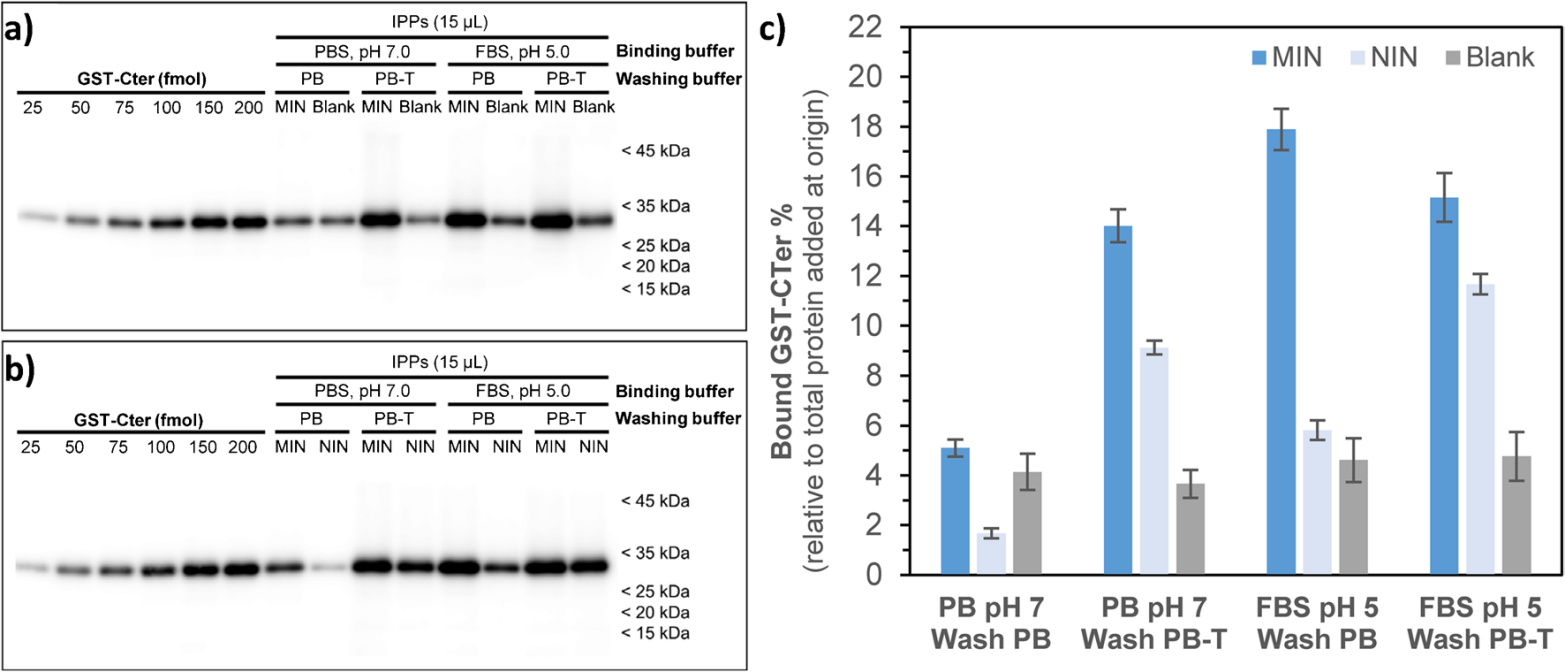
IPP assays to tune incubation buffer and washing conditions to achieve the highest specific to non-specific binding ratio. **a** Recombinant GST-CTer was loaded at depicted increasing amounts (25–200 fmol) (lanes 1–6) in parallel with 15 µL of IPP samples following binding assays conducted with 100 nM of the target protein and 0.015 mg mL^−1^ of MIN using different binding and washing buffers (lanes 7–14). Blank experiments were done in the absence of any nanoparticle. **b** Similar experiments to (**a**) but, here, results for MIN and NIN are depicted for comparison (lanes 7–14). **c** Quantification of the percentage of bound GST-CTer as a function of initial target protein concentration input. The data illustrate the binding efficiency of GST-CTer to MIN and NIN nanoparticles under different binding and washing conditions. Results are mean ± SD of *n* = 3 independent experiments

Initially, blank assays were performed in the absence of MIN to determine the best conditions for a minimal nonspecific GST-CTer to SMB binding. At these conditions, GST-CTer is not expected to be pulled down, as it is lacking a biotinylated ligand capable of interacting with SMB. However, under some of tested conditions, considerable non-specific binding between GST-CTer and streptavidin was observed, probably due to hydrophobic interactions. This was principally observed when using PBS, particularly at pH 7 (Fig. S6), which indicates that both pH and the presence of NaCl contribute to non-specific binding. This can be clearly deduced from the Western blot assay shown in Fig. S6a, which is relative to blank assays performed at different conditions. Here, immunoreactivity was observed in the samples incubated in PBS at pH 7. On the other hand, the results obtained after washings with or without Tween-20 suggested that the use of this detergent contributed to non-specific binding. Based on these observations, PB pH 7 and FBS pH 5 not including tween-20 were selected for subsequent IPP experiments focused on determining which conditions provided highest specific binding of MIN to GST-CTer. In this regard, a first series of IPP experiments was conducted using MIN nanoparticles alongside either blank experiments, in the absence of any nanoparticle (Fig. 6a), or using NIN as control (Fig. 6b). Here, it was intended to determine the best buffer (either PB pH 7 or FBS pH 5) and to evaluate the effect of adding Tween-20 to the washing step in achieving the highest specific to non-specific binding ratio. As shown in Fig. 6a, the lowest MIN/blank signal ratio was observed using PB (pH 7) for protein binding and washing, without the addition of Tween-20 (lanes 7–8). By contrast, the rest of tested conditions (lanes 9–15) were found to be more suitable for efficient GST-CTer IPP. To evaluate the specificity of MIN binding through imprinted sites, additional IPP experiments were performed by incubating the target protein with either MIN or NIN, with the later serving as a control (Fig. 6b). The use of Tween-20 in the washing buffer (PB-T) was counterproductive, as it failed to effectively remove non-specifically bound GST-CTer protein from NIN, resulting in much higher immunoreactivity (lanes 10 and 14) compared to those washed with PB (lanes 8 and 12). Consistent with these data, the percent of immunoprecipitated protein (Fig. 6c) showed that the best results for both MIN/blank and MIN/NIN ratios were obtained using FBS pH 5 as binding buffer and PB pH 7 for washing. Under these conditions, the signal resulting from IPP using NIN was comparable to the blank, which suggests that non-specific binding to the nanoparticles was minimal.

**Fig. 7:**
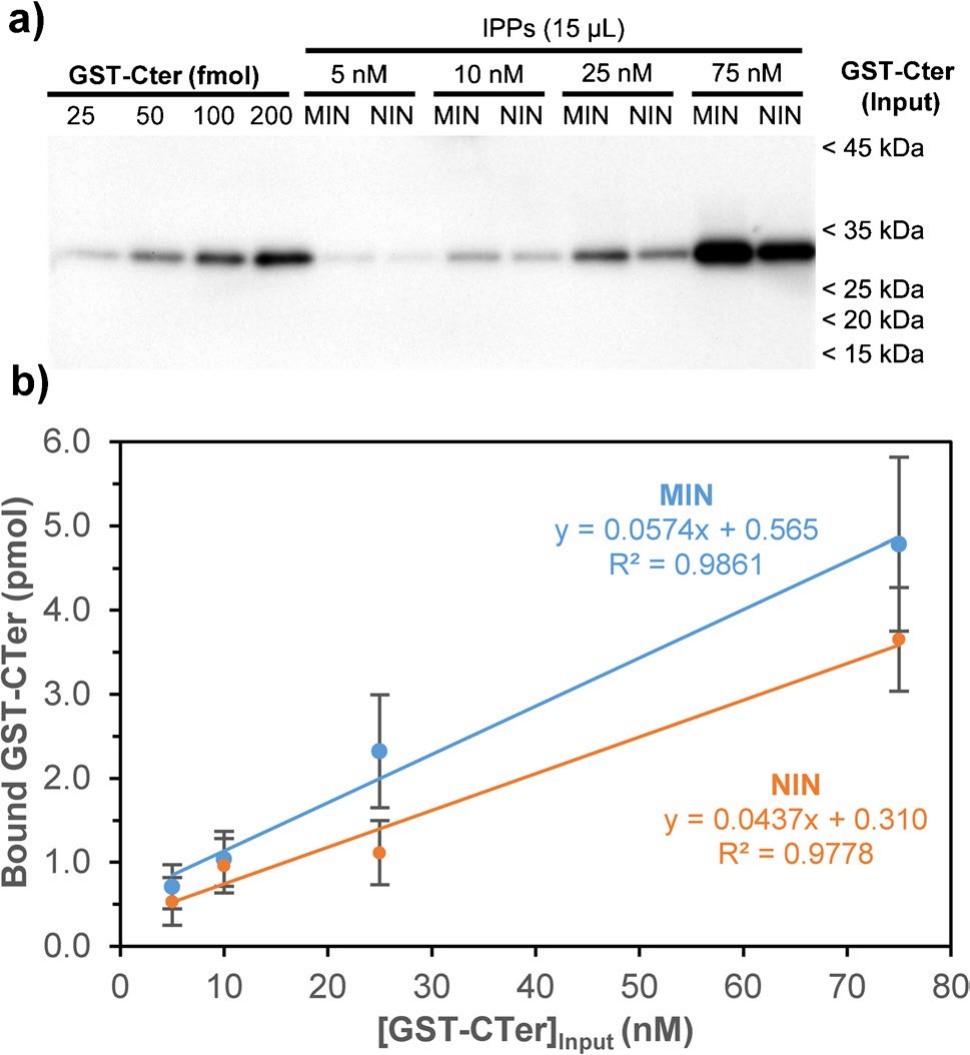
Immunoprecipitation and quantitative binding analysis of GST-CTer using MIN and NIN nanoparticles. **a** Immunoreactive signals detected by Western blot. Recombinant GST-CTer was loaded at increasing amounts (25–200 fmol) (lanes 1–4) in parallel with 15 µL of IPP samples following binding experiments with input protein concentrations ranging from 5 to 75 nM, incubated with MIN or NIN nanoparticles (lanes 5–12). **b** Quantification of bound GST-CTer (pmoles) measured as a function of initial input protein used for incubation with MIN or NIN. Results depict mean ± SD of *n* = 3 independent experiments

Further experiments were then conducted to assess the capability of anti-CB_1_ MIN to immunoprecipitate the target ligand at different concentrations. A linear increase of bound protein was observed with increasing GST-CTer input concentrations incubated with MIN or NIN (Fig. 7). Across all tested input concentrations, MIN yielded higher immunoprecipitation efficiency compared to NIN, with a slope of 0.0574 pmol nM^−1^, corresponding to an immunoprecipitation efficiency of 11.48% over the initial input concentration. This efficiency may be considered more than acceptable, since it exceeds by far the recovery threshold of 0.15% of input protein established by Venkataraman et al. [37].

**Fig. 8:**
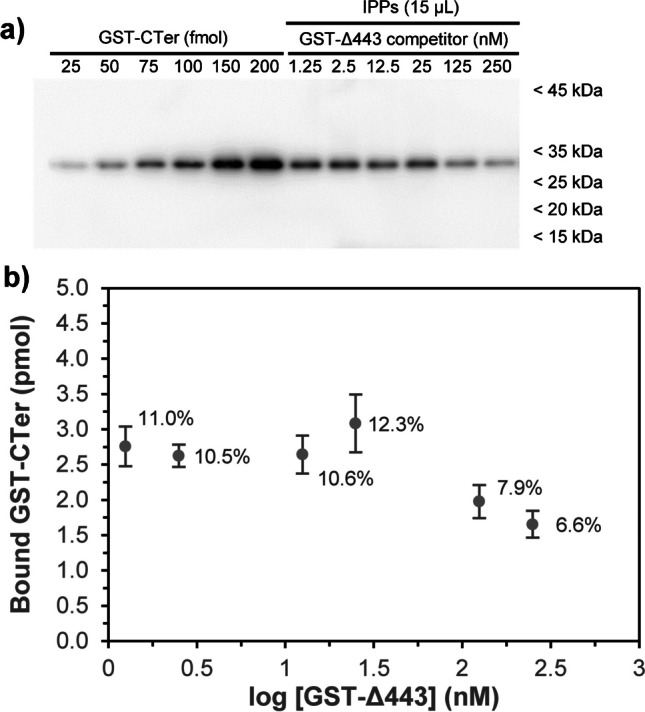
Competitive binding analysis using GST-Δ443 as a competitor for GST-CTer binding to MIN nanoparticles. **a** Recombinant GST-CTer was loaded at shown increasing amounts (lanes 1–6) in parallel with 15 µL of IPP samples following binding experiments with a fixed concentration of the target GST-CTer protein (50 nM) and increasing concentrations of the truncated fusion protein GST-Δ443 used as a competitor (lanes 7–12). **b** Quantification of bound GST-CTer (pmoles) as a function of the concentration of the competitor added to the incubation medium. Above each point, the calculated immunoprecipitated protein percentage relative to the initial input protein amount is depicted. Results depict mean ± SD of *n* = 3 independent experiments

Finally, a competition experiment was conducted using a fusion protein identical to the target GST-CTer protein (GST-CB1_414-472_) but truncated at amino acid 443 (GST-CB1_414-442_; GST-Δ443) as a competitor for the target protein. This protein lacks the 30 residues of the carboxy-end of the CB_1_ receptor [15, 38] and, consequently, the epitope sequence used for imprinting. It was hypothesised that GST-Δ443, devoid of the target amino acid sequence, would only bind to MIN through non-specific interactions outside imprinted sites being unable to compete with the target GST-CTer for binding sites. Therefore, the GST-Δ443 should provide little interference in the binding event of the target GST-CTer with imprinted sites created in MIN. To explore this, a fixed amount of MIN nanoparticles was mixed for 1 h with increasing concentrations of GST-Δ443, ranging from 1.25 to 250 nM, to block non-specific sites for the target GST-CTer protein. Then, the GST-CTer was added to achieve a final concentration of 50 nM, and it was incubated for 24 h at 37 °C under continuous orbital shaking as in previous IPP assays. As shown in Fig. 8a, the binding of the target GST-CTer to MIP nanoparticles remained apparently constant in the range of GST-Δ443 competitor concentrations between 1.25 and 25 nM (lanes 7–10), and only began to decrease at concentrations of the competitor of 125 nM, i.e. more than double than the GST-CTer target (lanes 11–12). Thus, quantitative analysis showed that about 10–12% of the initial GST-CTer input was immunoprecipitated after blocking with GST-Δ443 in the range between 1.25 and 25 nM, whereas the bound GST-CTer percentage decreased slightly to 7.9% and 6.6% after blocking with 2.5- and 5.0-fold GST-Δ443 relative to the target, respectively (Fig. 8b). This demonstrates that blocking non-specific sites has little influence on the binding event between the target protein and the synthesised anti-CB_1_ MIN.

## Conclusions

Biotinylated molecularly imprinted nanoparticles targeting the CB_1_ cannabinoid receptor have been explored here as potential antibody substitutes for protein immunoprecipitation. Produced nanoparticles respond to thermal stimuli, showing LCST values that change upon the cross-linker percentage used in the monomer mixture. Turbidity assays revealed that the cross-linker influences particle flexibility; however, binding capacity was not clearly compromised for most rigid nanoparticles, prepared with high cross-liker amounts. From batch rebinding experiments, it was concluded that ligand binding was maximised having the nanoparticles at the collapsed state (40 °C) at pH 5, conditions which were later tested for immunoprecipitation experiments. We wanted to explore immunoprecipitation as a new application using MIN as antibody substitutes, facing those MIN to GST-CTer recombinant fusion proteins. Upon optimised conditions, produced MIN were found capable to successfully pull-down the target ligand at different concentration levels. It was also observed that the truncated version of the recombinant protein, used as a competitor, did not compromise the specific MIN-GST-CTer binding, which corroborated that binding occurred preferably through the 458-KVTMSVSTDTSAEAL-472 epitope. Having examined the potential applicability of biotinylated artificial antibodies for protein immunoprecipitation, we may conclude that this application paves the way of MIN as artificial antibodies for protein extraction. This makes them particularly promising for extracting proteins such as the CB_1_ receptor from complex tissue homogenates, allowing for the removal of many interferences and simplifying protein quantification.

## Supplementary Information

Below is the link to the electronic supplementary material.

Supplementary file1 (DOCX 3987 KB)

Supplementary file2 (DOCX 8951 KB)

## Data Availability

No datasets were generated or analysed during the current study.
