# Supplementary material for: Tuning the properties of peptide imprinted nanoparticles for protein immunoprecipitation using magnetic streptavidin beads
Source: Mikrochim Acta. 2024 Oct 29;191(11):709. doi: 10.1007/s00604-024-06782-7 (PMC11522088; doi:10.1007/s00604-024-06782-7)
Supplement: Supplementary file 1 — Supplementary file1 (DOCX 3987 KB) [file 604_2024_6782_MOESM1_ESM.docx]

**Supplementary Material**

**Tuning the properties of peptide imprinted nanoparticles for protein immunoprecipitation using magnetic streptavidin beads**

Ainhoa Elejaga-Jimeno^1,4^, Alberto Gómez-Caballero^1,4^*, Gontzal García del Caño^2,5^, Nora Unceta^1,4^, Miquel Saumell-Esnaola^3,5^, Joan Sallés^3,5^, M. Aránzazu Goicolea^1,4^ and Ramón J. Barrio^1,4^.

^1^ Department of Analytical Chemistry, University of the Basque Country UPV/EHU, 01006 Vitoria-Gasteiz, Spain.

^2^ Department of Neurosciences, Faculty of Pharmacy, University of the Basque Country UPV/EHU, 01006 Vitoria-Gasteiz, Spain.

^3^ Department of Pharmacology, Faculty of Pharmacy, University of the Basque Country UPV/EHU, 01006 Vitoria-Gasteiz, Spain.

^4^ Bioaraba, MetaboloMIPs, 01008 Vitoria‑Gasteiz, Spain.

^5^ Bioaraba, Neurofarmacología Celular y Molecular, 01008 Vitoria‑Gasteiz, Spain.

^6^ Centro de Investigación Biomédica en Red de Salud Mental (CIBERSAM), 28029 Madrid, Spain.

*Corresponding author. Tel: +34-945013857

*E-mail address:* a.gomez@ehu.eus

**Experimental**

*Peptide labelling with Alexa Fluor*

In order to quantify the peptide via fluorescence spectrophotometry, it is necessary to label the ligand with a reagent that imparts an intense and stable fluorescent signal over time. One viable option for this purpose is to employ the fluorophore Alexa Fluor 448 C5 maleimide (Fig. S1). This compound can react with the -SH group of the cysteine additionally included to the 15 amino acid sequence of the target peptide (C-KVTMSVSTDTSAEAL) through the formation of a stable thioether linkage. Thanks to this reaction at the terminal cystein, the 15-amino acid fraction remains unaltered, being all 15 amino acids of the peptide available for interacting with produced molecularly imprinted nanoparticles (MIN).

The labelling protocol started dissolving 10.9 mg of the target peptide in 5 mL of a 0.1 M PBS buffer at pH 7.2, previously deoxygenated with N_2_. Subsequently, 1 mg of the Alexa-Fluor fluorophore, previously dissolved in 250 μL of DMSO, was added to the mixture. The mixture was left to react for 2 hours in the dark and under magnetic stirring. Following this, the fluorophore excess was removed through repeated dialysis in ultrapure water using a tubular membrane of Spectra/Por 6, with a nominal molecular weight of 1 kDa.

*Production of imprinted and control nanoparticles*

Polymer nanoparticles were fabricated by solid phase synthesis using the epitope imprinting approach [1]. A peptide having 15 amino acid and an additional cysteine (C-KVTMSVSTDTSAEAL) was immobilised on the surface of glass beads (GB), used as solid support. The immobilisation protocol is already described by our research group [2]. Briefly, GB were activated by boiling in concentrated NaOH, and then functionalised with the silane APTES. Next, the pendant amino groups were reacted with the cross-linker SIA, which introduces a terminal iodine for coupling the peptide in an oriented manner through the cysteine residue. As a result, a stable thioether linkage is created. Pierce BCA protein assay kit (Fisher Scientific, Spain) was used to estimate the amount of peptide bound to the surface of GB, finding 10.2±2.2 µg of peptide per g of GB. Finally, the peptide bound GB (GB/peptide) were washed repeatedly with 0.1 M PBS, water and ethanol.

30 g of GB/peptide were immersed in 25 mL of a 25 mM phosphate buffer solution (PB) at pH 7.4 to proceed with the solid-phase synthesis of polymer nanoparticles. The mixture was placed in a round bottom tube sealed with a rubber septum, and it was purged for 30 min with nitrogen for oxygen removal. Next, 69.5 mg of NIPAm (0.592 mmol), 67 mg of TBAm (0.511 mmol), 31 mg of APMA (0.168 mmol) and 6 µL of AA (0.087 mmol) were added as functional monomers. Furthermore, different amounts of the cross-linker BisAm were also included, that is, 11 mg, 23.5 mg, 37.5 mg, 53 mg or 70.5 mg to reach cross-linker percentages of 5%, 10%, 15%, 20% or 25%, respectively over the total moles of monomers. Finally, 14 mg of the water-soluble DCAA *iniferter* were added to the mixture and it was purged with nitrogen for another 5 min followed by three vacuum-nitrogen bubbling short cycles. For polymerisation, the tube containing the mixture was placed between two UV lamps and it was irradiated for 30 min. During the first 15 min, the mixture was kept in an ice bath while being irradiated, whereas last 15 min of polymerisation were performed at room temperature.

After synthesis, affinity based separation of MIN was done [2]. For this, MIN particles synthesised on the solid support (GB/Peptide/MIN) were transferred to a pre-fritted empty solid-phase extraction tube with a reservoir of 60 mL (Merck, Spain) and a porosity of 20 µm. Next, the tube was preconditioned in a water bath at 40ºC, and, then, 4x25 mL of ultrapure water were percolated to elute low affinity polymers and unreacted monomers. Thereafter, biotinylation of MIN remaining on the surface of GB was conducted, as they have pendant NH_2_ groups capable to react with NHS esters. To this end, 25 mL of PB containing 5 mg of biotin-NHS were added while the tube was maintained in a water bath at 40 ºC. The mixture was left to react for 2h and, then, another 4x25 mL of ultrapure water were percolated to remove unreacted biotin. In a final step, the tube was cooled in an ice bath, and high affinity MIN were eluted percolating 4x25 mL of borate buffer 0.1M (pH 9.5) at 6ºC achieving MIN suspension of 100 mL. This suspension was concentrated with a rotary evaporator first, and then, using Amicon Ultra-15 centrifugal filters (3 kDa NMWCO) from Merck (Spain). The final concentrate that remained in the Amicon filter was washed repeatedly with ultrapure water until conductivity decreased below 2 µS/cm in collected eluates.

Non imprinted nanoparticles (NIN), used as control nanoparticles, were produced using the same protocol as the above described but employing just bare GB, not having the target peptide covalently attached to their surface.

*Estimation of the biotinylation degree for imprinted and non-imprinted nanoparticles*

Biotinylation degree of the labelled polymer nanoparticles was determined with a Pierce Biotin Quantitation Kit (Thermo Scientific, USA). This kit contains a microtube having the 4'-hydroxyazobenzene-2-carboxylic acid (HABA)/Avidin coloured complex. 900 µL of 0.1M PBS buffer is added first to the microtube and absorbance is measured at 500 nm. Thereafter, 100 µL of the biotinylated MIN or NIN suspension is added to the tube and absorbance is recorded again. The biotin present on the nanoparticle surface binds to avidin and displaces HABA, which gives rise to an absorbance decrease. Based on this absorbance difference, the biotinylation degree can be estimated. The biotinylation degree for all produced nanoparticles is outlined in Table S1.

**Result and discussion**

*Nanoparticle response to thermal stimuli: LCST determination*

It is well known that polymer nanoparticles produced from monomers such as NIPAm and TBAm respond to thermal stimuli [2, 3], experiencing a volume phase transition from a hydrophilic coil state to a more hydrophobic globular state upon temperature increase over the LCST. As previously stated, the ability to respond to thermal stimuli depends on the cross-linker percentage used for nanoparticle synthesis. A higher cross-linker amount would *a priori* produce nanoparticles with less flexibility, thereby hindering nanoparticle shrinking and coil to globule transition. The work we are presenting here pretends to go further on this, exploring how the cross-linker influences physicochemical characteristics of produced nanoparticles, and, at the same time, evaluate how all this behaviour influences MIN binding to the target ligand. Accordingly, the initial experimental was focused on determining how MIN and NIN nanoparticles responded to thermal stimuli in order to know LCST values for each fabricated type of nanoparticle. LCST of the polymers was determined by turbidity experiments at 600 nm measuring absorbance of a 0.15 mg mL^-1^ MIN or NIN suspension as a function of temperature between 10 and 65 ºC. Recorded absorbance values for each MIN and NIN are depicted in Fig.S2. From these sigmoidal plots, the LCST of each polymer was determined at the point of 10% absorbance. Temperature-dependent absorbance change for MIN (Fig. 1a) or NIN (Fig. 1b) can be observed for nanoparticles synthesised using different cross-linker percentages. Concerning MIN, higher cross-linker percentages provide particles with apparently lower LCST. Besides, absorbance stabilisation, that is, the plateau of the graph, is achieved at lower temperatures when the cross-linking degree is higher. Coil to globule transition in MIN particles having 5% of cross-linker happens from 30 to 60 ºC, whereas for MIN having 25% of cross-linker it happens from 30 to 40 ºC, being much faster. This may be attributable to different flexibilities based on the cross-linker amount. In any case, all produced MIN showed thermoresponsiveness, even the ones apparently more rigid, produced with 25% of cross-linker. Higher cross-linker percentages than 25% gave rise to gelation of the whole polymerisation mixture, therefore, they were discarded for further experimental. Concerning NIN (Fig. 1b) differences between cross-linker percentages were not so evident. As a rule, LCST was also lower for NIN having more cross-linker, and coil to globule transition and stabilisation happened at a more narrow temperature interval. However, the tendency changed for NIN having 25% of cross-linker, probably because this percentage is close to the amount needed for solution gelation. All this may suggest that the absence of the template and binding sites created by itself provide NIN polymers with a more compact structure, less flexible and adaptable, resulting in lower overall critical temperatures. LCST values for all tested MIN and NIN are detailed in table 1.

**Supplementary figures**

**Fig. S1.** Peptide labelling reaction using Alexa Fluor C5 maleimide.


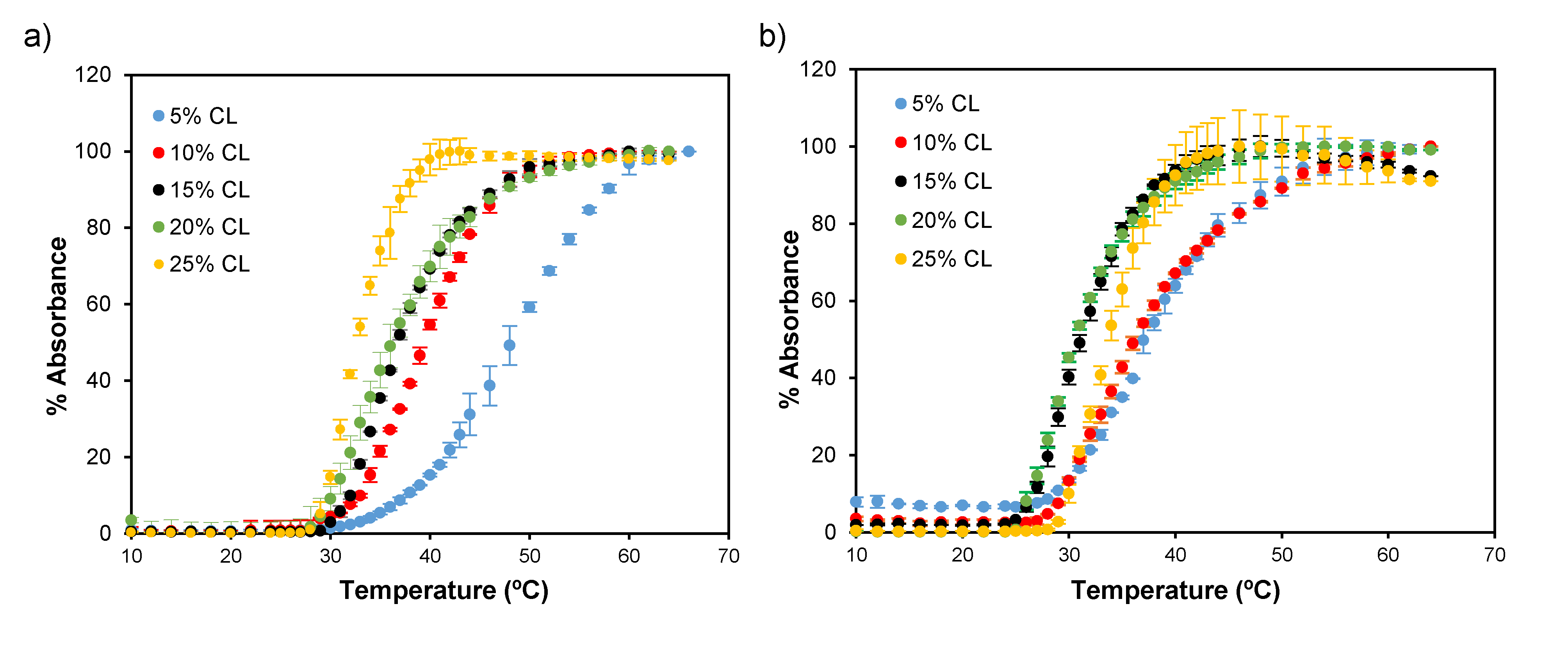


**Fig. S2.** Results obtained from turbidity tests showing relative absorbance measured at different temperatures for 150 mg L^-1^ suspensions of a) MIN or b) NIN nanoparticles in PBS 0.1 M at pH 7. Curves obtained using MIN and NIN nanoparticles synthesised with different cross-linker percentages are depicted.


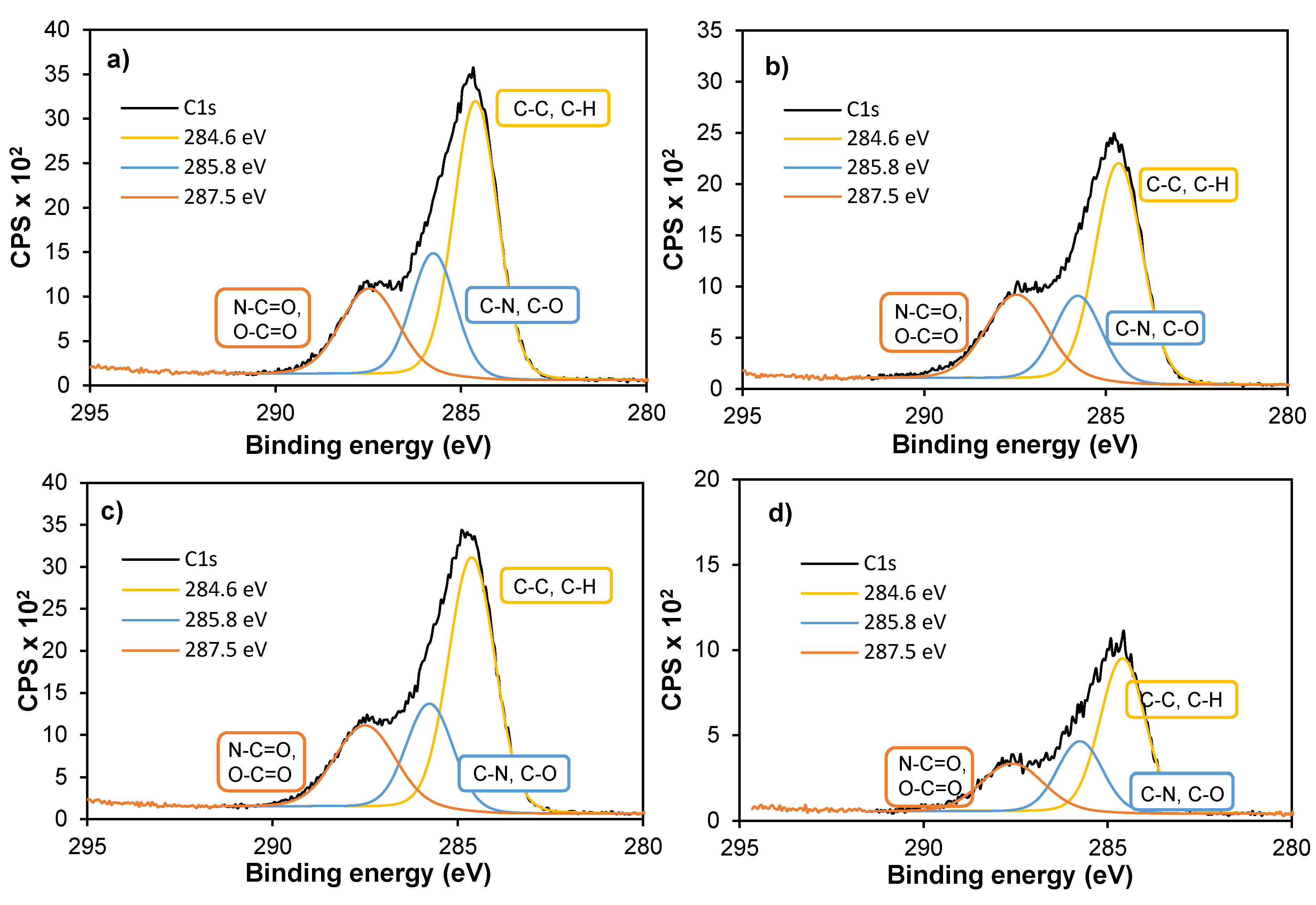


**Fig. S3.** XPS curve-fitting results of C1s for a) MIN, b) NIN, c) biotinylated-MIN and d) biotinylated-NIN.

**
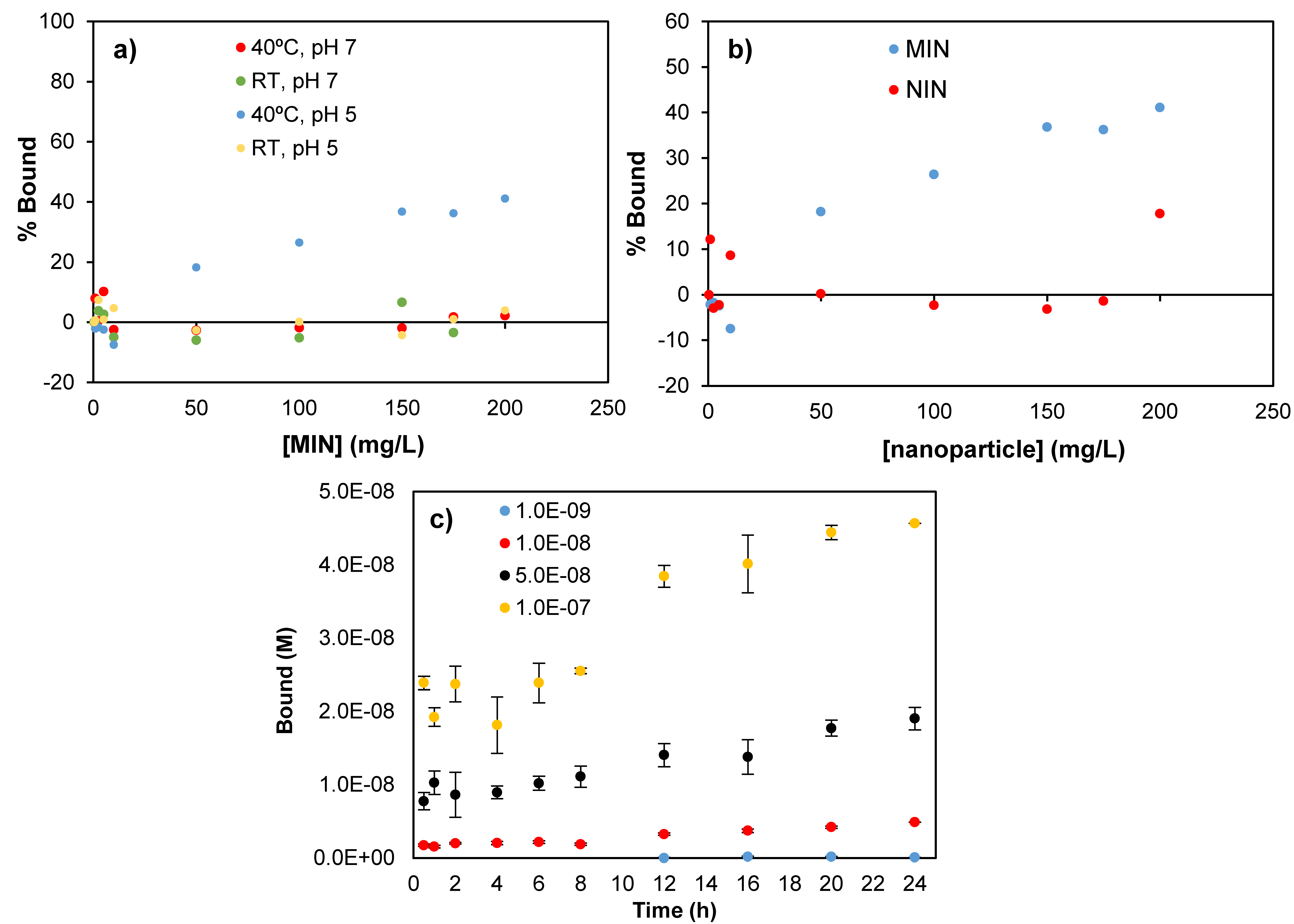
**

**Fig. S4.** Bound peptide dependence: a) on MIN concentration used for binding experiments at different conditions, b) on MIN or NIN nanoparticle concentration for binding experiments in 25 mM formate buffer (pH 5) at 40ºC, and c) on the incubation time used for binding experiments with peptide concentrations ranging from 10^-9^ to 10^-7^ M as ligand (n=3). MIN prepared with 10 % of CL were used for all these experiments.


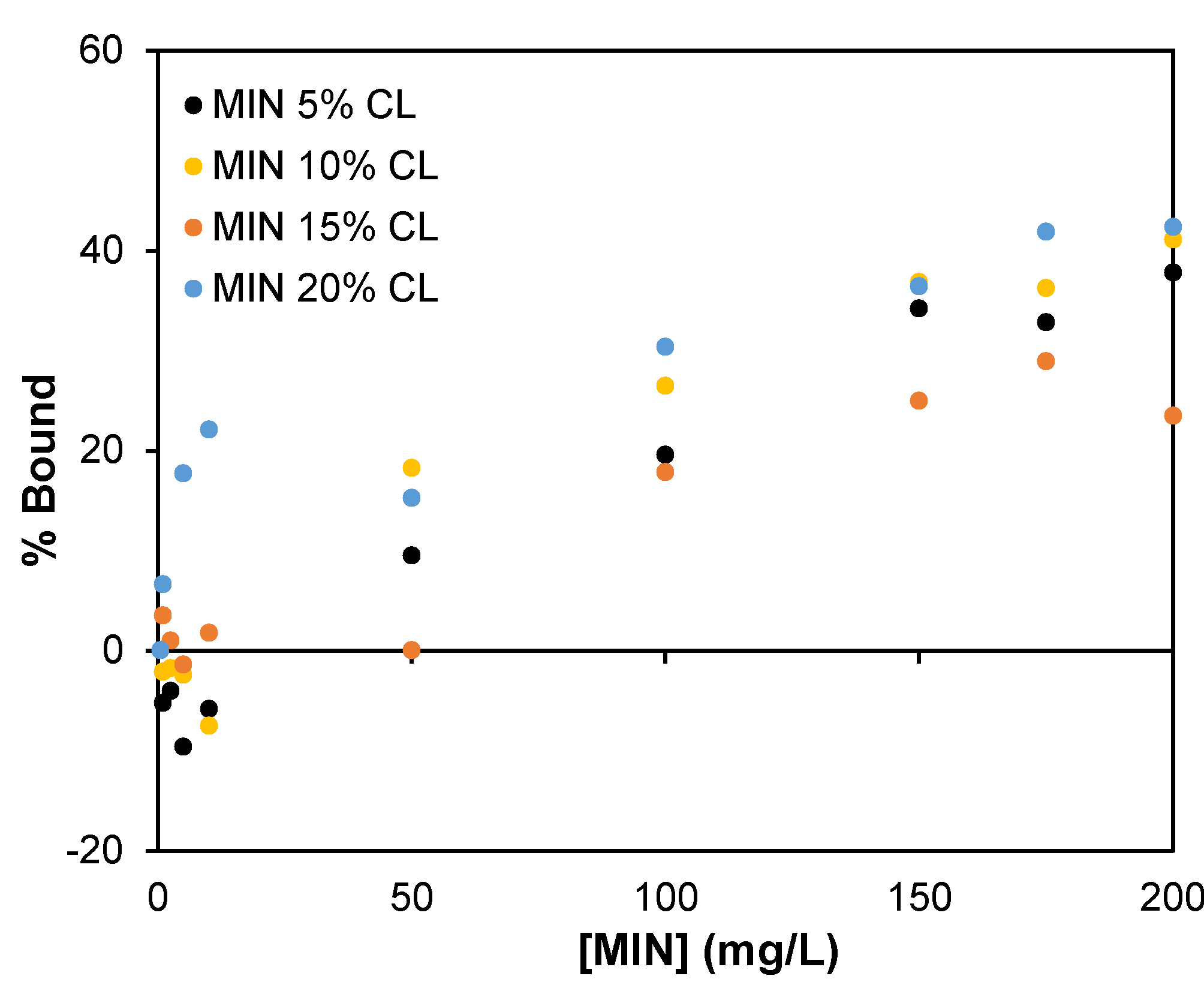


**Fig. S5.** Bound peptide percentage as a function of MIN nanoparticle concentration in binding experiments. MIN fabricated using different cross-linker percentages are depicted.


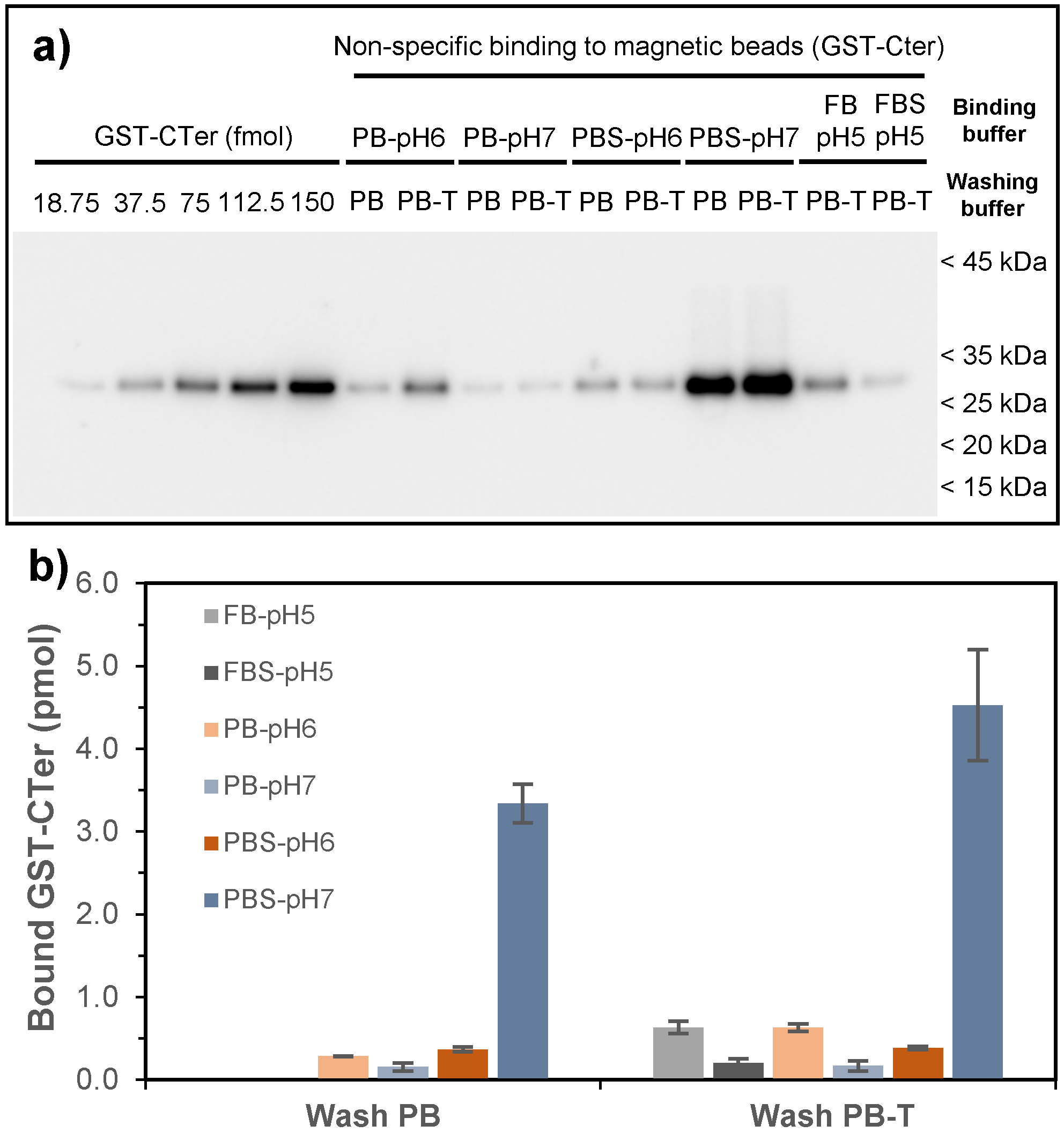


**Fig. S6.** Non-specific binding of GST-CTer to magnetic beads under various buffer conditions. a) Immunoreactive signals detected by Western blot. Recombinant GST-CTer was loaded at increasing amounts (lanes 1-5) in parallel with 15 µL of samples following binding experiments in the absence of any nanoparticles at a fixed concentration of target GST-Cter protein and different binding and washing buffer conditions (lanes 6-15). b) Quantification of bound GST-Cter (pmoles) as a function of initial target protein concentration input. Results are mean ± SD of three independent experiments (n=3).

**Supplementary tables**

**Table S1.** LCST values obtained from turbidity assays for each produced MIN and NIN nanoparticles.

|  |  | **Cross-linker (%)** | | | | |
| --- | --- | --- | --- | --- | --- | --- |
|  |  | **5 %** | **10 %** | **15 %** | **20 %** | **25 %** |
| LCST (⁰C) | **MIN** | 37.2 ± 0.9 | 32.3 ± 0.3 | 30.9 ± 0.1 | 29.3 ± 1.2 | 29.0 ± 0.4 |
|  | **NIN** | 26.8 ± 0.1 | 27.8 ± 0.1 | 25.9 ± 0.2 | 25.4 ± 0.3 | 29.7 ± 0.1 |

**Table S2.** Biotinylation degree of MIN and NIN nanoparticles expressed as µg of biotin / mg of polymer

|  | **MIN**  (µg of biotin / mg polymer) | **NIN**  (µg of biotin / mg polymer) |
| --- | --- | --- |
| **5% CL** | 14.43 ± 0.06 | 5.86 ± 0.04 |
| **10% CL** | 9.90 ± 0.05 | 2.44 ± 0.01 |
| **15% CL** | 23.22 ± 0.03 | 22.25 ± 0.02 |
| **20% CL** | 24.44 ± 0.03 | 16.41 ± 0.04 |
| **25% CL** | 12.25 ± 0.02 | 19.21 ± 0.02 |

**Table S3.** Summary of XPS results obtained for biotinylated and non biotinylated MIN and NIN polymers.

| **Sample** | **Element** | **Assignment** | **Binding energy (eV)** | **% At Conc** | **% At rel** |
| --- | --- | --- | --- | --- | --- |
| **MIN** | C | C-C, C-H | 284.6 | 38.97 | 72.0 |
|  |  | C-N, C-O | 285.8 | 17.27 |  |
|  |  | N-C=O, O-C=O | 287.5 | 15.72 |  |
|  | O | O 1s | 531.1 | 12.56 | 15.8 |
|  |  | O 1s | 532.5 | 3.23 |  |
|  | N | N 1s | 399.5 | 10.9 | 12.2 |
|  |  | N 1s | 401.1 | 1.34 |  |
| **NIN** | C | C-C, C-H | 284.6 | 38.94 | 73.3 |
|  |  | C-N, C-O | 285.8 | 15.05 |  |
|  |  | N-C=O, O-C=O | 287.5 | 19.35 |  |
|  | O | O 1s | 531.1 | 11.64 | 13.8 |
|  |  | O 1s | 532.9 | 2.16 |  |
|  | N | N 1s | 399.5 | 11.16 | 12.9 |
|  |  | N 1s | 401.6 | 1.72 |  |
| **MIN-biotin** | C | C-C, C-H | 284.6 | 38.57 | 70.6 |
|  |  | C-N, C-O | 285.8 | 15.91 |  |
|  |  | N-C=O, O-C=O | 287.5 | 16.08 |  |
|  | O | O 1s | 531.1 | 13.86 | 17.1 |
|  |  | O 1s | 532.6 | 3.27 |  |
|  | N | N 1s | 399.5 | 10.7 | 12.1 |
|  |  | N 1s | 401.2 | 1.42 |  |
|  | S | S 2p | 163.4 | 0.19 | 0.2 |
| **NIN-biotin** | C | C-C, C-H | 284.6 | 39.45 | 72.4 |
|  |  | C-N, C-O | 285.7 | 17.28 |  |
|  |  | N-C=O, O-C=O | 287.5 | 15.69 |  |
|  | O | O 1s | 531.0 | 12.28 | 14.8 |
|  |  | O 1s | 532.5 | 2.52 |  |
|  | N | N 1s | 399.4 | 11.06 | 12.8 |
|  |  | N 1s | 401.0 | 1.53 |  |
|  | S | S 2p | 162.9 | 0.19 | 0.19 |

**References**

1. Teixeira SPB, Reis RL, Peppas NA, et al (2021) Epitope-imprinted polymers: Design principles of synthetic binding partners for natural biomacromolecules. Sci Adv 7:. https://doi.org/10.1126/sciadv.abi9884

2. Gómez-Caballero A, Elejaga-Jimeno A, García del Caño G, et al (2021) Solid-phase synthesis of imprinted nanoparticles as artificial antibodies against the C-terminus of the cannabinoid CB1 receptor: exploring a viable alternative for bioanalysis. Microchim Acta 188:. https://doi.org/10.1007/s00604-021-05029-z

3. Gómez-Caballero A, Unceta N, Goicolea MA, Barrio RJ (2021) Plastic Receptors Developed by Imprinting Technology as Smart Polymers Imitating Natural Behavior. In: Reactive and Functional Polymers Volume Three. Springer International Publishing, Cham, pp 69–116
