# Supplementary material for: Tuning the properties of peptide imprinted nanoparticles for protein immunoprecipitation using magnetic streptavidin beads
Source: Mikrochim Acta. 2024 Oct 29;191(11):709. doi: 10.1007/s00604-024-06782-7 (PMC11522088; doi:10.1007/s00604-024-06782-7)
Supplement: Supplementary file 2 — Supplementary file2 (DOCX 8951 KB) [file 604_2024_6782_MOESM2_ESM.docx]

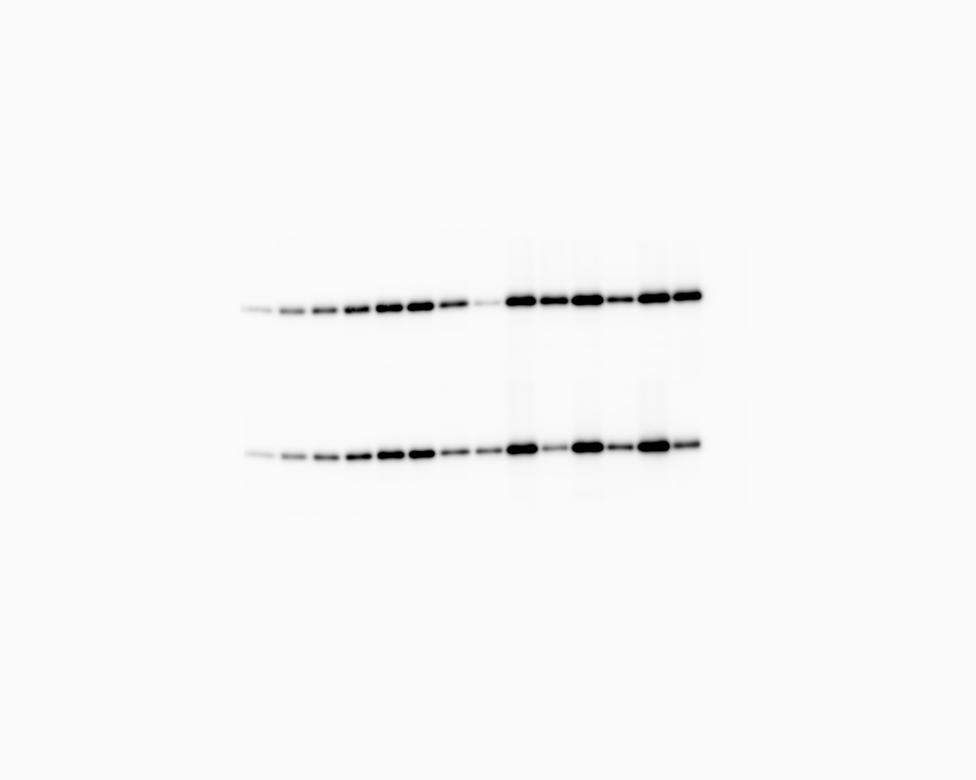


Uncropped image corresponding to immunoblots shown in Figs. 6a (lower blot) and 6b (upper blot) developed at once.


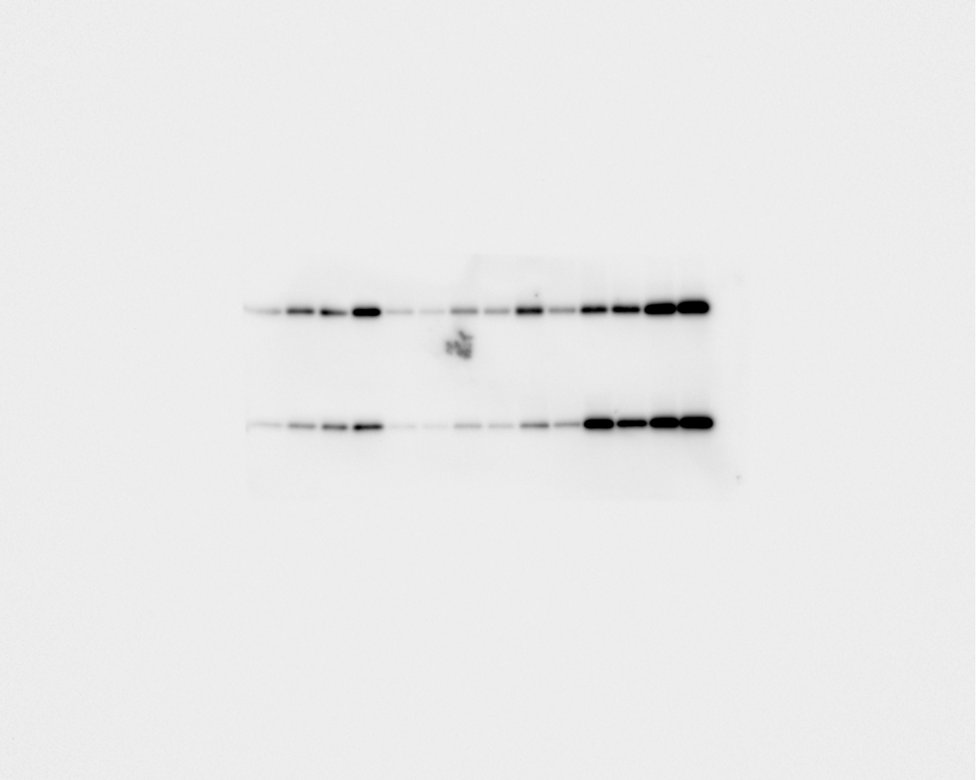


Uncropped images of a duplicate corresponding to the immunoblot shown in Fig. 7. The last two lanes (13-14) were loaded with purified GST-Cter to prevent the eventual diffusion of proteins in lane 12.


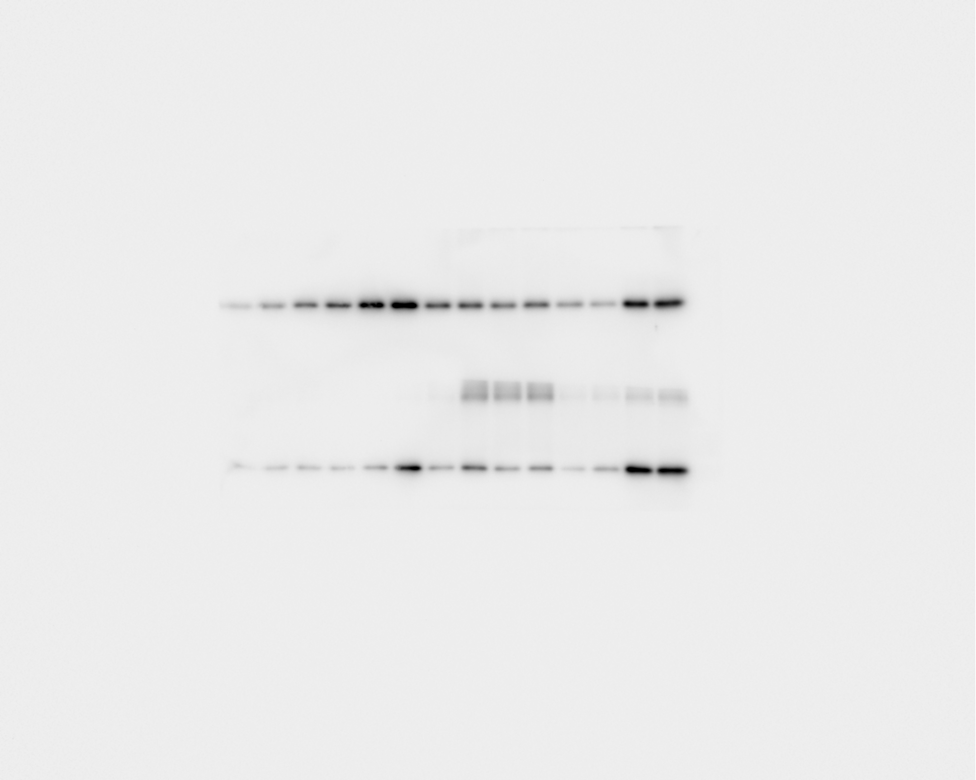


Uncropped images of a duplicate corresponding to the immunoblot shown in Fig. 8. The last two lanes (13-14) were loaded with purified GST-Cter to prevent the eventual diffusion of proteins in lane 12.


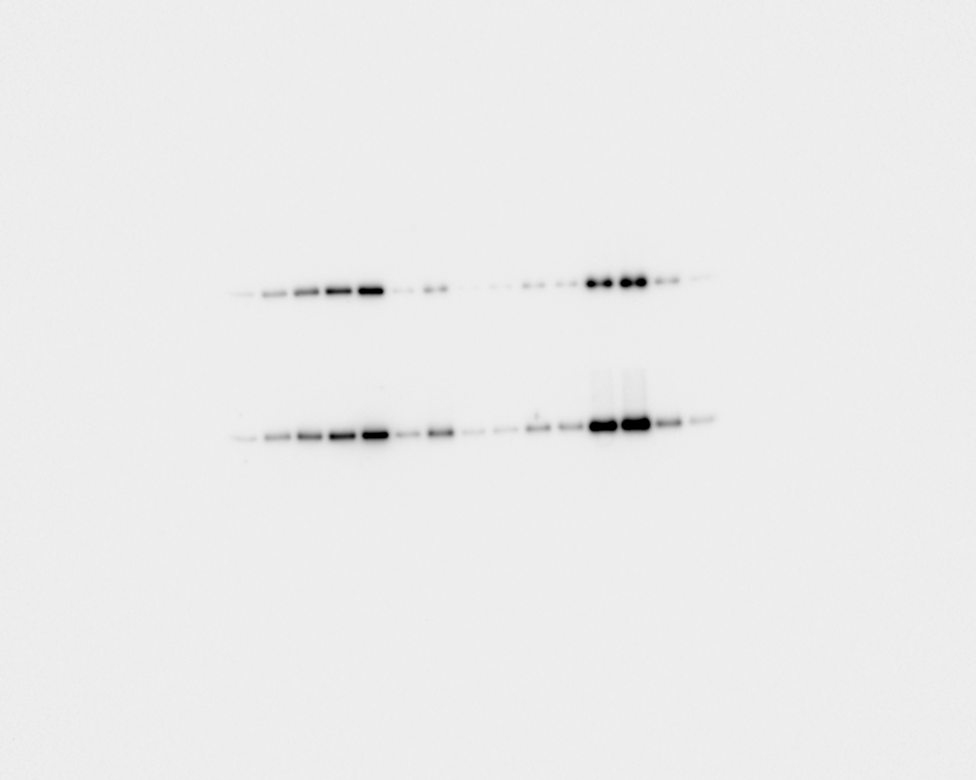


Uncropped images of a duplicate corresponding to the immunoblot shown in Fig. S6.
